# Differential splicing of neuronal genes in a Trem2*R47H mouse model mimics alterations associated with Alzheimer’s disease

**DOI:** 10.1186/s12864-023-09280-x

**Published:** 2023-04-04

**Authors:** Ravi S. Pandey, Kevin P. Kotredes, Michael Sasner, Gareth R. Howell, Gregory W. Carter

**Affiliations:** 1 The Jackson Laboratory for Genomic Medicine, 10 Discovery Drive, Farmington, CT 06032 USA; 2 The Jackson Laboratory, 600 Main Street, Bar Harbor, ME 04609 USA

**Keywords:** Genetics, Alzheimer’s disease, Mouse models, Genomics, Splicing, Transcriptomics

## Abstract

**Background:**

Molecular characterization of late-onset Alzheimer’s disease (LOAD), the leading cause of age-related dementia, has revealed transcripts, proteins, and pathway alterations associated with disease. Assessing these postmortem signatures of LOAD in experimental model systems can further elucidate their relevance to disease origins and progression. Model organisms engineered with human genetic factors further link these signatures to disease-associated variants, especially when studies are designed to leverage homology across species. Here we assess differential gene splicing patterns in aging mouse models carrying humanized *APOE4* and/or the Trem2*R47H variant on a C57BL/6J background. We performed a differential expression of gene (DEG) and differential splicing analyses on whole brain transcriptomes at multiple ages. To better understand the difference between differentially expressed and differentially spliced genes, we evaluated enrichment of KEGG pathways and cell-type specific gene signatures of the adult brain from each alteration type. To determine LOAD relevance, we compared differential splicing results from mouse models with multiple human AD splicing studies.

**Results:**

We found that differentially expressed genes in Trem2*R47H mice were significantly enriched in multiple AD-related pathways, including immune response, osteoclast differentiation, and metabolism, whereas differentially spliced genes were enriched for neuronal related functions, including GABAergic synapse and glutamatergic synapse. These results were reinforced by the enrichment of microglial genes in DEGs and neuronal genes in differentially spliced genes in Trem2*R47H mice. We observed significant overlap between differentially spliced genes in Trem2*R47H mice and brains from human AD subjects. These effects were absent in *APOE4* mice and suppressed in *APOE4*.Trem2*R47H double mutant mice relative to Trem2*R47H mice.

**Conclusions:**

The cross-species observation that alternative splicing observed in LOAD are present in Trem2*R47H mouse models suggests a novel link between this candidate risk gene and molecular signatures of LOAD in neurons and demonstrates how deep molecular analysis of new genetic models links molecular disease outcomes to a human candidate gene.

**Supplementary Information:**

The online version contains supplementary material available at 10.1186/s12864-023-09280-x.

## Background

Late-onset Alzheimer’s disease (LOAD) is the leading cause of dementia but has uncertain etiology. Recent characterization of molecular changes associated with AD pathology from postmortem brain studies have identified abundant alterations in transcripts, proteins, and processes, including differential gene splicing. In a recent study, deep proteomic analysis of brain tissue identified alternations in abundance of RNA binding proteins, suggesting a potential role for aberrant RNA splicing in AD pathogenesis [1]. Transcriptome-wide analysis of aging prefrontal cortex of human subjects identified genetic variants and *trans* acting splicing factors involved in dysregulation of mRNA splicing affecting brain transcriptomes in Alzheimer’s disease [2]. In a second study, proteomic profiles of Alzheimer’s disease brains identified an increased aggregation of insoluble U1 snRNP, a small nuclear RNA (snRNA) component of the spliceosomal complex, suggesting that the core splicing machinery may be altered in Alzheimer’s disease [3]. With this growing list of disrupted splicing events in AD brains, it provides a great opportunity to investigate the factors that contribute to mis-splicing events, understand how they lead to disease and conceive ways to correct pathological splicing through targeted therapeutics, and use splice variations as robust biomarkers.

Animal models provide the experimental platforms to study disease-relevant molecular changes in vivo, including alternative splicing, and enable translation through pre-clinical testing [4, 5]. Mouse models have been developed to understand and treat splicing defects associated with many diseases including AD and tauopathies [6–8]. In a previous study, neuron-specific alternative splicing of amyloid precursor protein (APP) was identified in brain tissue of hAPP transgenic mice, suggesting a role of splicing defects of hAPP in the development of AD-type brain changes [9]. However, these studies have largely focused on alternative splicing of selected candidate genes, and investigations of global splicing events in LOAD mouse models are scarce. Using transcriptome-wide analyses of new mouse models, we recently found upregulation of the spliceosome pathway in an aging mouse model carrying the AD risk-associated R47H point mutation in the *Trem2* gene [10]. This work motivated us to further investigate alternative splicing events in these mouse models and identify possible functional consequences that represent AD pathogenesis.

Trem2 (triggering receptor expressed on myeloid cells 2) is a receptor protein expressed in dendritic cells, macrophages, and microglia either on the plasma membrane, intracellularly, or as a soluble factor [11, 12]. Viewed primarily as an immune responsive factor, when expressed on the plasma membrane Trem2 binds to phospholipids, apolipoproteins, and DNA to activate pathways related to cytokine and chemokine production, motility, survival, and phagocytosis [13]. Downstream signaling leads to alterations in microglia activation states and neuroinflammation [14]. In the brain, amyloid beta has also been shown to bind to Trem2 on homeostatic microglia responsible for sensing and clearing the neural environment [15]. In the context of the central nervous system, mutations to Trem2 are associated with serious human disease. The R47H mutation in *Trem2* is one of the strongest genetic risk factors of late-onset Alzheimer’s disease (LOAD) [16–19]. However, following CRISPR-mediated insertion of the R47H point mutation into humanized-*Trem2* mice, we noticed a novel, cryptic splice acceptor site in Exon 2 that is not observed in humans. The resultant splice variant leads to a pronounced decrease in expression. Since, we have generated additional, humanized Trem2*R47H alleles that produce normal levels of transcript in the brains of mouse hosts. Characterization and comparison of these strains is ongoing which will provide insight into the consequences of this human disease risk factor on mouse health. It is well described in humans that increased inflammation follows with increasing age [20] and is associated with development of systemic and neurological pathologies. Thus, it is possible that a reduction in Trem2 expression is protective against AD progression via reduced neuroinflammation. However, microglia sensing, pruning, and clearing of apoptotic neurons is also highly dependent on Trem2 receptors and justifies more attention in the developmental context of AD [21].

Alternative splicing is an important post-transcriptional regulatory mechanism through which multiple mRNAs are produced from a single pre-mRNA molecule. Alternative splicing is a major contributor to cellular diversity in both normal tissues and disease. Alternative splicing affects over 95% of mammalian genes [22, 23], contributing significantly to the functional diversity of proteins expressed in tissues [24]. Tissue specific alternative splicing events tend to be associated with specific functions of the tissue. For instance, brain-specific alternative splicing events are often associated with neural-specific functions [24]. Alternative splicing is abundant in human nervous system tissues and contributes to phenotypic differences within and between individuals [25]. The mechanisms underlying altered splicing involve either disruption of *cis*-acting elements within the affected gene and/or *trans*-acting factors (e.g., RNA-binding proteins) that are required for normal splicing or splicing regulation such as the heterogeneous ribonucleoproteins (hnRNPs) [4, 26]. Splicing defects can cause disease, modify the severity of disease phenotypes, or correspond to disease susceptibility [4, 26]. There are many diseases that result from disrupted alternative splicing, such as amyotrophic lateral sclerosis (ALS), autism, and cancer [4, 27]. Disruptions in mRNA splicing are also associated with age-related disorders, such as frontotemporal lobar dementia (FTD) [28], Parkinson’s disease [29] and Alzheimer’s disease [9, 30].

Here, by applying multiple analytic methods, we investigated genome-wide splicing events in new LOAD mouse models created by knocking-in human *APOE4* sequence and the R47H mutation in *Trem2*. We annotated the differentially spliced genes using functional and cell type enrichment analyses. Moreover, we compared the splicing events in the LOAD mouse models with splicing events reported in human AD studies [1–3]. We identified a number of differential splicing events in mouse models that mimic results from human AD studies, including differential isoform usage in the heterogeneous ribonucleoprotein M (hnRNPM). In order to assess the effects of isoform switch in hnRNPM, we investigated the binding sites of disrupted RNA-binding proteins on other differentially spliced genes in the LOAD mice. Overall, we identified numerous splicing alterations in our *Trem2.R47H* mouse model that were also observed in human AD cohorts, suggesting that the mechanisms of AD risk involve splice alterations in neurons.

## Results

**Fig. 1:**
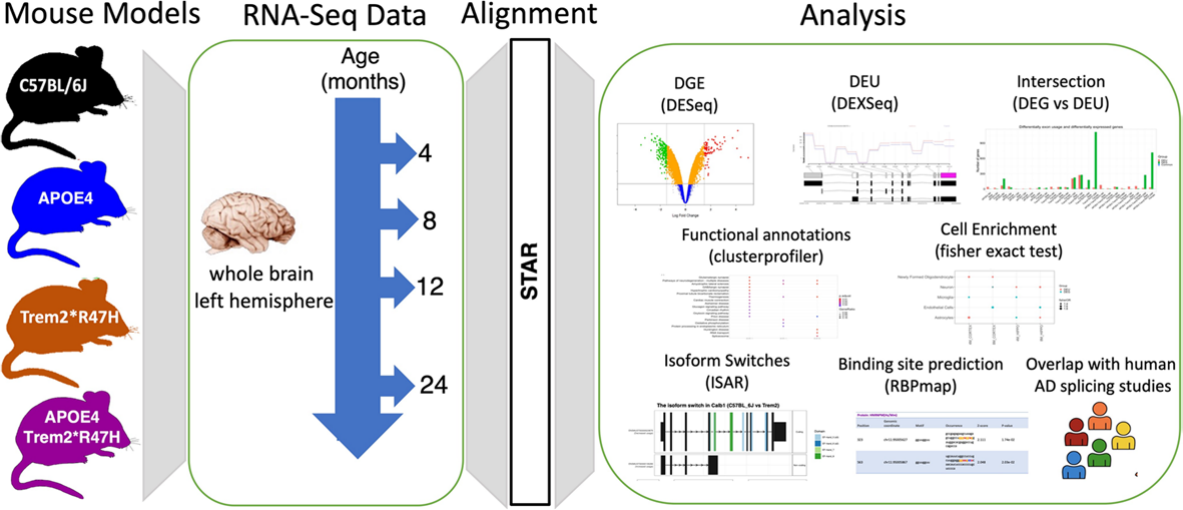
Schematic overview of the analysis. Bulk RNA-Seq data from mouse whole brain hemispheres were processed by our parallelized RNA-Seq analysis pipeline. We performed differential gene expression analysis, differential exon usage analysis, and differential isoform usage analysis. After performing differential analysis, we used the software clusterProfiler to perform functional enrichment analysis followed by cell type enrichment analysis. The RBPmap webserver was used to predict Binding sites of RNA-binding proteins on differentially spliced genes. In addition, we performed hypergeometric tests to identify enrichment of differentially spliced gene set in human Alzheimer’s cases

Differential gene expression and functional analyses of the brain transcriptomes identified age-dependent molecular changes associated with AD pathologies in the *APOE4*, Trem2*R47H and APOE4.Trem2*R47H mice on a C57BL/6J background [10]. Notably, in 12-month-old Trem2*R47H male mice, significantly increased expression was observed for genes associated with the spliceosome pathway, suggesting the possibility of differential splicing events in this mouse model [10]. This effect was not observed in double-mutant APOE4.Trem2*R47H mice. These findings motivated us to further investigate alternative splicing events in the same panel of mouse models that might correspond to AD pathogenesis in humans. Here we systematically investigate the splicing events in mouse models (Fig. 1; Supplementary Fig. 1).

### Differentially spliced genes were enriched in neuronal functions associated with AD

**Fig. 2:**
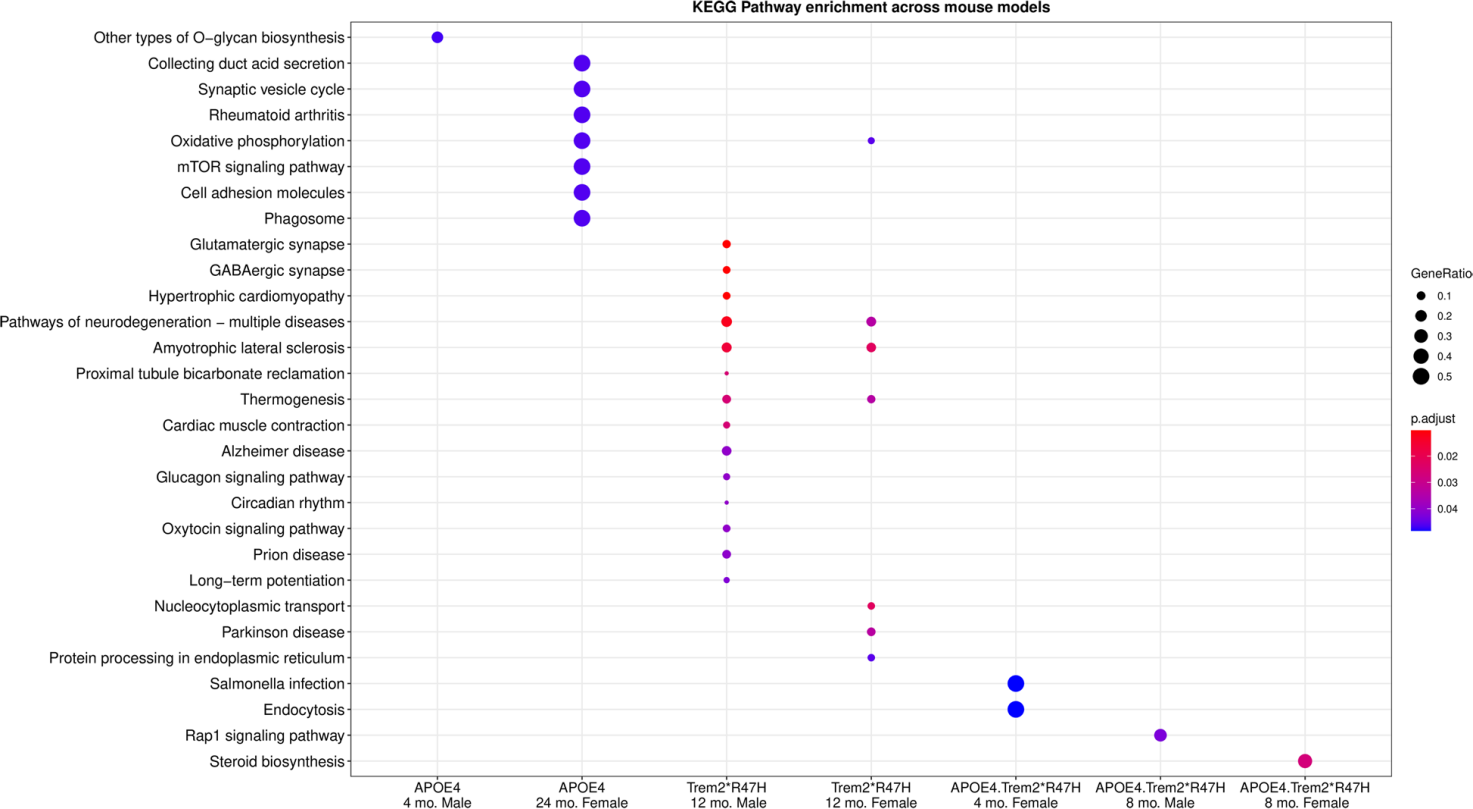
Functional Analysis of Differentially Exon Usages (DEU) genes. Significantly enriched KEGG pathways (*p* < 0.05) in the set of DEU genes across all mouse models using enrichKEGG function build under clusterprofiler R package (mo. indicates months)

First, we applied DEXSeq [31] on processed transcriptomic data to identify genes with differential exon usages (DEU) to infer differential splicing events in the LOAD mouse models compared to age and sex-matched B6 controls. The greatest number of DEU genes were observed in Trem2*R47H mice at 12 months of age, which agrees with differential expression patterns and corresponds to the age at which spliceosome gene abundances were altered [10]. In this strain, we identified a total of 50 genes with significant DEU (p_adj_ < 0.1) at 4 months, 71 genes with significant DEU at 8 months (p_adj_ < 0.1), 480 genes with significant DEU (p_adj_ < 0.1) at 12 months, and 50 genes with significant DEU (p_adj_ < 0.1) at 24 months (Table 1; Supplementary Table 1). Functional analysis of these DEU genes returned multiple AD associated biological processes (Supplementary Table 2). Interestingly, DEU genes in 12-month-old Trem2*R47H male mice were enriched for neuron-specific process such as ‘GABAergic synapse’ and ‘glutamatergic synapse’ (Fig. 2; Supplementary Table 2), suggesting perturbation in microglial gene *Trem2* leads to differential splicing in genes expressed in neurons.

**Table 1:** Differentially exon usages genes in mouse models used in this study. Number of genes with differential exon usage in each mouse model compared to age and sex-matched B6 control mice. Number in parentheses shows the overlap between genes with DEU and differentially expressed genes (DEGs)

MOUSE MODELS	AGE (in months)	Genes with differential exon usage (DEU)	
		**MALE**	**FEMALE**
**APOE4**	4	40 (0)	22 (0)
	8	69 (3)	49 (1)
	12	4 (1)	6 (1)
	24	12 (0)	12 (0)
Trem2*R47H	4	9 (0)	41 (0)
	8	35 (0)	36 (0)
	12	207 (8)	273 (9)
	24	23 (0)	27 (2)
APOE4.Trem2*R47H	4	77 (0)	11 (0)
	8	46 (0)	13(0)
	12	48 (1)	56 (1)
	24	20 (0)	31 (2)

Relative to Trem2*R47H brains, DEU was lower in both *APOE4* and APOE4.Trem2*R47H mice. In humanized *APOE4* mice, we identified a total of 62 genes with significant DEU (p_adj_ < 0.1) at 4 months, 118 genes with significant DEU (p_adj_ < 0.1) at 8 months, 10 genes with significant DEU (p_adj_ < 0.1) at 12 months, and 24 genes with significant DEU (p_adj_ < 0.1) at 24 months (Table 1; Supplementary Table 1). Functional analysis showed enrichment for DEU genes involved in ‘synaptic vesicle cycle’, and ‘mTOR signaling’ related pathways in 24-month-old *APOE4* female mice (Fig. 2; Supplementary Table 2). In APOE4.Trem2*R47H mice, we observed a total of 89 genes with significant DEU (p_adj_ < 0.1) at 4 months, 58 genes with significant DEU (p_adj_ < 0.1) at 8 months, 104 genes with significant DEU (p_adj_ < 0.1) at 12 months, and 51 genes with significant DEU (p_adj_ < 0.1) in 24-month-old mice (Table 1; Supplementary Table 1). Functional analysis showed enrichment for DEU genes involved in ‘endocytosis’, and ‘protein processing in ER’ related pathways in 8 months old APOE4.Trem2*R47H mice, respectively (Fig. 2; Supplementary Table 2). The reduction of DEU in APOE4.Trem2*R47H mice relative to Trem2*R47H mice recapitulates the DEG patterns previously observed [10] and further supports a suppression of mid-life Trem2*R47H mutation effects by the presence of humanized *APOE4*.

**Fig. 3:**
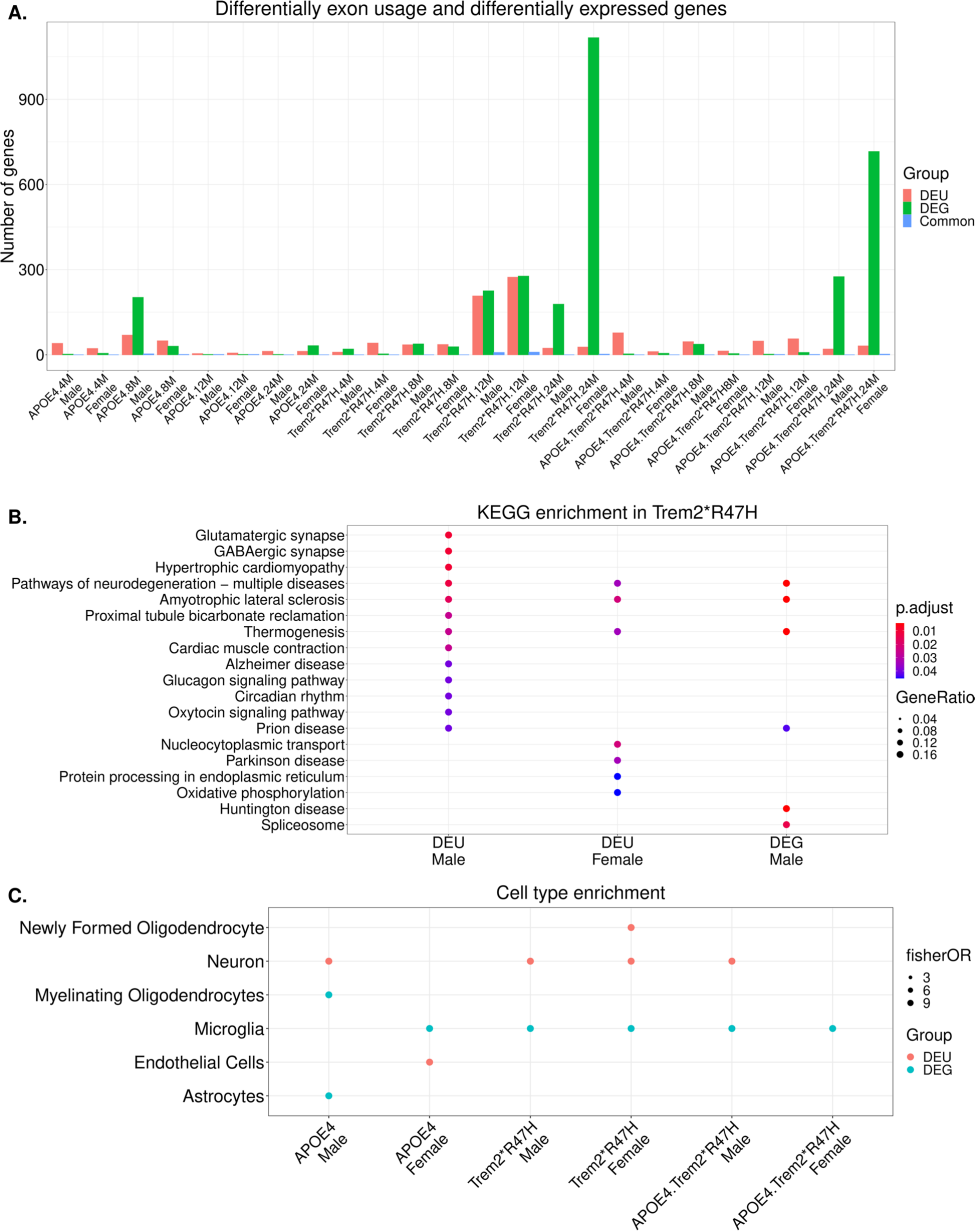
Differential splicing analysis using DEXSeq. **A** Number of genes with differential exon usage (DEU) and differential expressed genes (DEG) in each mouse model. Common infers gene overlap between DEG and DEU genes. **B** Significantly enriched KEGG pathways in the set of differential exon usage (DEU) and differentially expressed genes (DEG) in the 12 months old Trem2*R47H mice (*p* < 0.05). **C** Cell type enrichment analysis in the gene set with differential exon usage (DEU) and differentially expressed genes (DEG) in each mouse model using Fisher exact test. Significant cell type enrichment (*p* < 0.1) are shown by solid dots

Next, we performed a comparative analysis between differentially spliced genes and differentially expressed genes across all mouse models. We did not observe any significant overlap between sets of DEU genes and differentially expressed genes (DEGs) for any mouse models (Fig. 3A; Supplementary Table 3). Furthermore, in 12 months old Trem2*R47H male mice, differentially expressed genes were enriched for RNA splicing related functions [10], whereas DEU genes were enriched for neuronal related functions (Fig. 3B; Supplementary Table 2). To better understand the difference between DEGs and DEU genes, we evaluated enrichment of cell-type specific genes [32] in these gene sets for each mouse model. We identified that DEU genes in Trem2*R47H and APOE4.Trem2*R47H male mice were enriched in neuronal signatures, while DEGs were enriched in the microglial signatures (Fig. 3C). In *APOE4* male mice, DEU genes were enriched in the neuronal signatures, while DEGs were enriched for the astrocytes and the myelinating oligodendrocytes signatures. DEU genes in the *APOE4* female mice were enriched for microglial cell type signatures, while DEGs were enriched in the endothelial cells (Fig. 3C). Overall, we identified more alternatively spliced genes in 12-month-old Trem2*R47H mice compared to other LOAD mouse models, which were further enriched for neuronal associated functions.

### Identification of alternative splicing events in *Trem2* knockout model suggest possible role of *Trem2* gene in disrupting AD through alternative splicing mechanism

The Trem2*R47H mouse model used in this study exhibits both expression of the R47H mutation and expression levels reduced by approximately 50% [10]. To investigate whether differential splicing events are due to altered expression of *Trem2* gene or specific to R47H mutation in *Trem2* gene, we performed differential splicing analysis using DEXSeq on processed transcriptomic data from cortex and hippocampus brain regions of *Trem2* Knockout (KO) and respective WT mice from a recent study [33]. Since this study assessed only male mice, we compared all results from *Trem2* KO mice with *Trem2.R47H* male mice.

Interestingly, we identified more DEU genes in *Trem2* KO mice compared to Trem2*R47H mice, even at younger ages (Table 2; Supplementary Table 4). Next, we computed the intersection between DEU genes in *Trem2* KO mice and 12-month-old *Trem2.R47H* mice and found significant gene overlaps (*p* < 0.05, hypergeometric test). Combined across both tissues, 56 DEU genes were common between 4-month-old *Trem2* KO and 12-month-old *Trem2.R47H*, and 55 DEU genes were common between 8-month-old *Trem2* KO and 12-month-old *Trem2.R47H* (Table 2). We did not observe a significant overlap between DEU genes in 4-month-old Trem2*R47H mice and *Trem2* KO mice (Table 2), suggesting perturbations in splicing start earlier in mice with *Trem2* deletions compared to mice carrying the R47H mutation in *Trem2*.

**Table 2:** Differentially spliced genes in *Trem2* KO mice. Number of genes showing differential exon usage in *Trem2* KO male mice compared to respective B6 control mice in Cortex and hippocampus brain regions. Overlap between DEU genes in *Trem2* KO male mice and Trem2*R47H male mice at 4, 8, 12, and 24 months. Significance of overlaps were computed using hypergeometric test

Brain Tissue	Age (in months)	DEU Genes in Trem2.KO mice	Overlapped with DEU genes in Trem2.R47H mice			
			**12 months Trem2.R47H**	**4 mo. Trem2.R47H**	**8 mo. Trem2.R47H**	**24 mo. Trem2.R47H**
**Cortex**	4	734	38 (*p* = 4.29E-13)	1 (*p* = 0.36)	5 (*p* = 0.02)	2 (*p* = 0.28)
	8	874	34 (*p* = 1.51E-08)	2 (*p* = 0.09)	3 (*p* = 0.30)	1 (*p* = 0.72)
**Hippocampus**	4	782	26 (*p* = 1.50E-05)	1 (*p* = 0.37)	4 (*p* = 0.09)	4 (*p* = 0.02)
	8	723	30 (*p* = 4.29E-13)	2 (*p* = 0.07)	2 (*p* = 0.48)	1 (*p* = 0.65)

Similar to *Trem2.R47H* mice, we did not observe any significant overlap between DEGs and DEU genes in *Trem2* KO mice (Supplementary Fig. 2A). Functional analyses revealed enrichment of ‘synaptic vesicle cycle’ and ‘protein processing in ER’ pathways in DEU genes in hippocampus brain region of 8 months old *Trem2* KO mice (Supplementary Fig. 2B; Supplementary Table 2). Further, DEGs are mostly enriched for microglial and astrocytes signatures, while DEU genes are enriched for neuronal and oligodendrocytes and astrocytes cell signatures in *Trem2* KO mice (Supplementary Fig. 2C). All together, these results suggest that differential splicing is due to reduced expression of *Trem2* gene possibly combined with a loss-of-function from the *Trem2.R47H* variant, as we observed similar pattern of splicing events (enriched in neuronal signatures) in *Trem2* KO mice models as *Trem2.R47H*.

### Differential spliced genes in human AD cases significantly overlapped with differentially spliced genes in the *Trem2.R47H* mice

We next compared our findings with results from multiple studies of alternative splicing events in human Alzheimer’s disease populations [1–3]. In these studies, different approaches were employed to assess aberrant mRNA splicing events in distinct human AD cohorts. We computed overlaps between gene sets identified as differentially spliced in LOAD mouse models and differentially spliced genes reported in human AD cases [1–3].

In the first study, Towfique et al. identified 84 genes with alternative splicing events related to AD by measuring differential intron usages in human AD cases compared to controls [2]. We identified 67 genes out of the 84 with mouse orthologs (Methods). Cell enrichment analysis identified that these 67 genes were significantly enriched for newly formed oligodendrocytes cell signatures (*p* < 0.1) (Supplementary Fig. 3). Next, we found that 12 out of the 67 genes were identified as differentially spliced (genes with DEU) across all our mouse models, representing a significant overlap (hypergeometric *p* = 0.004). Notably, 8 of these 12 genes (*ENO2, LUC7I3, KIF21A, MAP4K4, PPP3CA, MACF1, NDRG4,* and *PRRC2C*) were differentially spliced in 12-month-old Trem2*R47H mice, which was highly significant (hypergeometric *p* = 2.78 × 10^–6^) (Table 3). We did not observe any significant overlap with any other mouse models.

**Table 3:** Summary of overlap between differentially spliced genes identified in multiple human AD splicing studies and mouse models. Number of differentially spliced genes identified in splicing analyses of human AD cohort in multiple studies. Significant overlap with differentially exon usage genes in mouse models were computed using hypergeometric test (*p* < 0.05). Control-AsymAD represents comparison between asymptomatic AD cases and controls. AsymAD-AD represents comparison between AD cases and asymptomatic AD cases. Control-AD represents comparison between AD cases and controls

	**Differentially Spliced Genes**	**Overlap with 12 mo. *Trem2* male mice** **(207 DEU)**	**Overlap across all mouse models** **(1167 DEU)**
**ROSMAP**	67	8 (*p* = 2.78E-06)	12 (*p* = 0.004)
**TMT Proteomic Study** **(Control-AsymAD)**	155	6 (*p* = 0.017)	27 (*p* = 0.001)
**TMT Proteomic Study (AsymAD-AD)**	291	10 (*p* = 0.005)	34 (*p* = 0.10)
**TMT Proteomic Study** **(Control-AD)**	237	5 (*p* = 0.20)	26 (*p* = 0.18)
**Emory/Kentucky**	536	13 (*p* = 0.03)	55 (*p* = 0.01)

In the second study, Johnson et al. investigated RNA splicing events by predicting alternative exon-exon junction splicing events across all subjects (symptomatic AD (*n* = 20), asymptomatic AD (*n* = 14), and control (*n* = 13) cases) [1]. For comparison, we extracted mouse orthologs for 237 proteins identified with differential alternative exon-exon junction in human symptomatic AD cases compared to controls (Control-AD); 155 proteins with differential alternative exon-exon junction in human asymptomatic AD cases compared to controls (Control-AsymAD); and 291 proteins with differential alternative exon-exon junction in human symptomatic AD cases compared to asymptomatic AD cases (AsymAD-AD) (Table 3). Cell enrichment analysis identified that these genes were enriched in oligodendrocytes (*p* < 0.05) (Supplementary Fig. 3). We found that 10 DEU genes *(ENO2, KIF21A, ATL3, GLS, STXBP1, PPP3CA, MACF1, MAP1B, DPP6*, and *SMS*) in 12-month-old Trem2*R47H mice overlapped with differentially spliced genes identified in human AD cases compared to AsymAD cases (hypergeometric *p* = 0.005) (Table 3). Similarly, 5 genes *(EFTUD2, DNM1L, STXBP1, DPP6*, and *SMS*) with DEU in 12-month-old Trem2*R47H mice overlapped (hypergeometric *p* = 0.2) with differentially spliced genes in human AD cases compared to controls; and 6 genes (*EFTUD2, KIF21A, DNM1L, MACF1, IST1,* and *PPP1R9B*) with DEU in 12 months old Trem2*R47H mice overlapped (hypergeometric *p* = 0.02) with differential spliced gene sets in human asymptomatic AD cases compared to controls (Table 3). We identified more DEU genes in 12-month-old Trem2*R47H mice overlapped with human AsymAD-AD group compared to Control-AsymAD group, which could be due to the fact that more differential splicing events were identified in human symptomatic AD cases compared to asymptomatic AD cases. Two genes *EFTUD2* and *DNM1L* were differentially spliced in both asymptomatic and symptomatic AD cases compared to controls, while two genes *DPP6* and *SMS* were differentially spliced in symptomatic AD cases compared to both asymptomatic AD cases and controls.

In the third study, Bai et al. investigated proteomic profiles of Alzheimer’s disease brains and identified accumulation of 36 proteins including insoluble U1-70 K and other small nuclear ribonucleoprotein spliceosome components, suggesting a possible loss of nuclear spliceosome activity in Alzheimer’s disease [3]. In this study 631 genes were identified with differential splicing deficiency scores in AD cases. We identified 536 genes out of the 631 with mouse orthologs (Methods). Further, cell enrichment analysis identified enrichment of neuronal cell signatures in these 536 genes with differential splicing efficiency in human AD cases (*p* < 0.05) (Supplementary Fig. 3). A total of 55 genes out of 536 genes with differential splicing efficiency in human AD cases were also identified as DEU genes across all mouse models (*p* = 0.01) (Table 3). Thirteen of these 55 genes overlapped with DEU genes in 12-month-old Trem2*R47H male (*p* = 0.03) and 14 genes with Trem2*R47H female mice (*p* = 0.07) (Table 3). Next, 4 of these 55 genes (*GPR107, EFTUD2, MAN1C1, and GRSF1*) overlapped (*p* = 0.03) with DEU genes in 8 months old Trem2*R47H male mice. We did not observe any significant overlap with any other mouse models.

In summary, two genes (*ENO2 and PPP3CA*) that were identified as DEU in 12-month-old Trem2*R47H male mice were identified as differentially spliced in all three human studies [1–3], while *STXBP1 and EFTUD2* were identified as differentially spliced in Johnson et al. and Bai et al. [1, 3]. Moreover, two genes (*KIF21A* and *MACF1*) that overlapped among 12-month-old Trem2*R47H male mice, human Control-AsymAD, and AsymAD-AD group were also identified as differentially spliced in prefrontal cortex of AD cases by Towfique et al. [2]. *MACF1* is a F-actin binding protein, and suggested to play role in AD pathogenesis as it is critical for neuron migration, neurite formation, synaptic function, and hippocampus-dependent learning and memory [34]. *KIF21A* is a family member of *KIF21B* and has similar amino acid composition. Kif21A is expressed throughout neurons, while *KIF21B* is mainly expressed in dendrites [35, 36]. Both *KIF21A* and *KIF21B* are linked to several human diseases. Enhanced *KIF21B* expression has been associated with severe AD pathology [35], while *KIF21A* is linked to congenital fibrosis of the extraocular muscles type 1 (CFEOM1), a disease characterized by absence of motor neurons of the midbrain [37]. Together, this suggests that aberrant splicing in *KIF21A* may disrupt neuronal functions associated with AD*.*

### Differential isoform usage analyses identified isoform switch in RNA binding proteins associated with splicing mechanism

We identified multiple genes with differential exon usages, specifically in 12-month-old Trem2*R47H mice, using DEXSeq [31]. This approach pinpoints the location of the difference by focusing on specific exons, but it is also informative to determine the resulting transcripts with altered abundances. Therefore, we have implemented IsoformSwitchAnalyzeR (ISAR) to identify isoform switches (reduced abundance of one isoform and increased abundance of another isoform of a given gene) and predict potential functional consequences of the identified isoform switches such as loss/gain of protein domains, signal peptides, and intrinsically disordered regions [38].

We identified many genes with differential isoform switches across LOAD mouse models (Table 4). We observed the greatest number of differential isoform switches in 12-month-old Trem2*R47H male (256 isoform switches across 215 genes) and 24-month-old Trem2*R47H female mice (267 isoform switches across 218 genes) (Table 4; Supplementary Table 5). Hereafter, we refer to genes with at least one differential isoform switch as genes with differential transcript usages (DTU). In order to identify if genes with differential isoform usage are with differential used exons, we computed overlap between DTU and DEU genes and found only 6 genes (*Ppp1r9b, Celf4, Fars2,Gabra1, Safb, and Pgbd5*) common between both gene sets for 12-month-old Trem2*R47H male mice, and 4 genes (*Morf4l2, Mcoln1, Atp2a2, and Usp18*) common between both gene sets for 12-month-old Trem2*R47H female mice (Table 5). For other mouse models either none or only one gene were common between DTU and DEU genes. We identified few genes with differential isoform usages that also has differentially used exons, suggesting either changes in single exons may be compensated by changes in other exons for a given isoform, or isoform usage might be driven by sub-significant changes in multiple exons. Hence, we further investigated genes with differential isoform switches in order to identify more signal associated with alternative splicing that might be missed by DEU analyses.

**Table 4:** Differentially isoform usages genes identified in mouse models using IsoformSwitchAnalyzerR (ISAR). Number of genes showing differential isoform (or transcript) usage in each mouse model compared to age and sex-matched B6 control mice

Mouse Models	Age	Sex	All Isoform with differential usage		
			**nrIsoforms**	**nrSwitches**	**nrGenes**
**APOE4**	4	Male	66	81	58
	4	Female	77	88	63
	8	Male	54	66	49
	8	Female	39	51	35
	12	Male	62	86	57
	12	Female	45	65	40
	24	Male	28	34	24
	24	Female	36	39	27
Trem2*R47H	4	Male	54	60	47
	4	Female	36	41	31
	8	Male	54	65	45
	8	Female	56	61	50
	12	Male	273	256	215
	12	Female	94	91	77
	24	Male	21	24	17
	24	Female	276	267	218
APOE4.Trem2*R47H	4	Male	88	106	77
	4	Female	39	46	31
	8	Male	62	72	55
	8	Female	46	50	39
	12	Male	41	49	34
	12	Female	78	99	72
	24	Male	18	23	15
	24	Female	41	44	34

**Table 5:** Overlap between DEGs, DTU, and DEU genes in Trem2*R47H mice models. Number of genes showing differential expression (DEGs), differential exon usage (DEUs), and differential isoform usage (DTUs) in Trem2*R47H mice at 12 and 24 months compared to age and sex-matched B6 control mice and genes overlapped between three groups

	**Differentially expressed genes** **(DEGs)**	**Differentially transcript usage genes** **(gDTU)**	**Differentially exon usage genes** **(gDEU)**	**Overlap between DEGs and gDTU**	**Overlap between gDTU and gDEU**
**12-month-old Trem2 male mice**	206	215	207	7	6
**12-month-old Trem2 female mice**	285	77	274	3	4
**24-month-old Trem2 male mice**	144	17	24	0	0
**24-month-old Trem2 female mice**	748	218	28	27	0

**Fig. 4:**
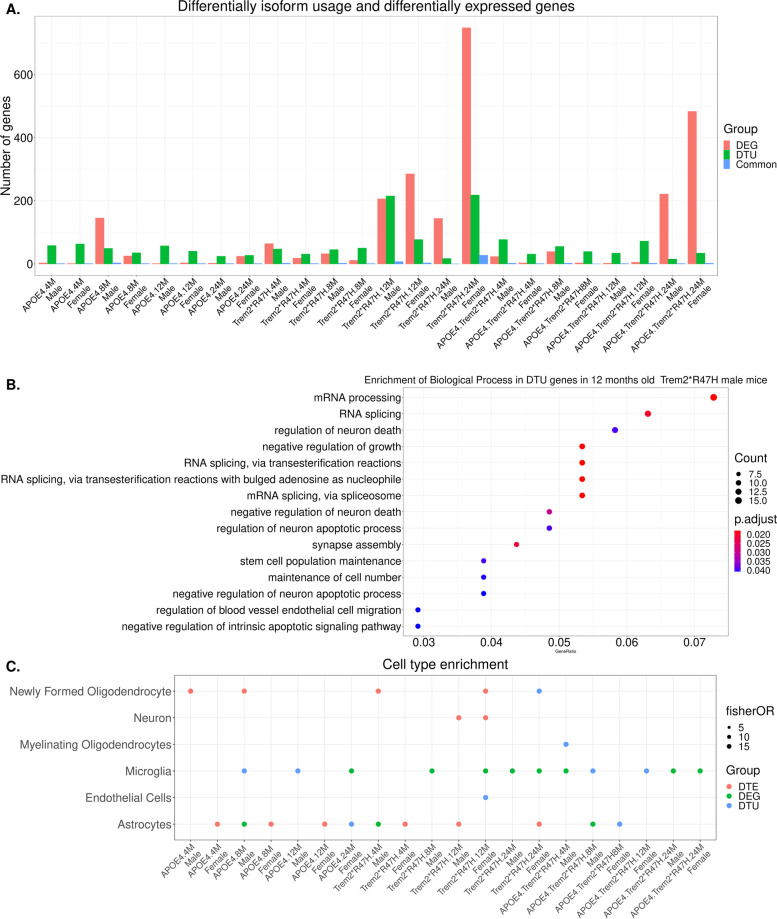
Differential splicing analysis using IsoformSwitchAnalyzerR (ISAR). **A** Number of genes with differential transcript usage (DTU) and differential expressed genes in each mouse model. Common infers gene overlap between DEG and DTU genes. **B** Significantly enriched biological process in the set of differential transcript usage (DTU) in the 12 months old Trem2*R47H mice (*p* < 0.05). **C** Cell type enrichments in the gene set with differential exon usage (DEU) and differentially expressed genes (DEG) in each mouse model using Fisher exact test. Significant cell type enrichment (*p* < 0.1) are shown by solid dots

First, we computed the intersection between DTU genes and differentially expressed genes (DEGs) across all mouse models. As with DEU genes, we did not observe any significant overlap between DTU genes and DEGs for any mouse model (Fig. 4A; Supplementary Table 3). Further, cell enrichment analysis determined that DTU genes in 12-month-old Trem2*R47H mice were not significantly enriched for neuronal signatures like DEU genes (Fig. 4B). However, functional analyses identified enrichment of splicing and neuronal related biological process in DTU genes in 12-month-old Trem2*R47H male mice (Fig. 4C; Supplementary Table 6), while DTU genes in 12-month-old Trem2*R47H female mice were enriched for protein folding and catabolic associated processes (Supplementary Table 6).

To compare with differentially spliced genes reported in human AD cohorts [1–3], we found that DTU genes in 12-month-old Trem2*R47H male mice showed significant overlap with differentially spliced genes reported in Johnson et al. and Bai et al. [1, 3]. A total of 7 genes out of 215 DTU genes in 12-month-old Trem2*R47H male mice overlapped (hypergeometric *p* = 0.004) with differentially spliced genes in human asymptomatic AD cases compared to controls (*PPP1R9B, TMED4, HYOU1, NT5DC1, PFN2, CCDC91,* and *PSMD2*), symptomatic AD cases compared to controls *(EIF5B, HNRNPM, CDK5, NPEPPS, KIF3A, MRPS7,* and *CANX*), and symptomatic AD cases compared to asymptomatic AD cases *(YWHAE, SPTBN1, CDK5, MGLL, NT5DC1, PFN2,* and *MAGI2*) [1] (Table 6). Further, 15 genes *(SUB1, TCF25, NDUFV1, R3HDM2, CALM2, CALB1, PUM2, PFN2, ACTN2, ARHGEF3, SLMAP, CELF4, MED28, FARS2,* and *PSMD2*) were found common (hypergeometric *p* = 0.003) between DTU genes in 12-month-old Trem2*R47H male mice and differentially spliced genes reported in Bai et al. [3] (Table 6). On the other hand, we found only one gene (*Clu*) common between DTU genes in 12-month-old Trem2*R47H male mice and differentially spliced genes reported in Towfique et al. [2] (Table 6).

**Table 6:** Summary of differentially spliced genes identified in multiple human AD splicing studies and overlap with mouse models. Number of differentially spliced genes identified in splicing analyses of human AD cohort in multiple studies and overlap with differentially spliced genes in mouse models were identified using differential isoform usage approach (IsoformSwitchAnalyzerR). Control-AsymAD represents comparison between asymptomatic AD cases and controls. AsymAD-AD represents comparison between AD cases and asymptomatic AD cases. Control-AD represents comparison between AD cases and controls

	**Differentially Spliced Genes**	**Overlap with 12 mo. *Trem2* male mice** **(215 DTU)**	**Overlap across all mouse models** **(1410 DTU)**
**ROSMAP**	67	1 (*p* = 0.58)	4 (*p* = 0.83)
**TMT Proteomic Study** **(Control-AsymAD)**	155	7 (*p* = 0.004)	16 (*p* = 0.23)
**TMT Proteomic Study (Asym-AD)**	291	7 (*p* = 0.08)	16 (*p* = 0.97)
**TMT Proteomic Study** **(Control-AD)**	237	7 (*p* = 0.03)	17 (*p* = 0.79)
**Emory/Kentucky**	536	15 (*p* = 0.003)	44 (*p* = 0.6)

Next, we investigated DTU genes enriched for splicing associated functions (Fig. 4C; Supplementary Table 2) and identified five of these genes (*Hnrnpm, Celf5, Snrpb2, Bcl2l2, and Rbm39*) with RNA binding domains; i.e. these genes encode for RNA-binding proteins (RBPs). Interestingly, the HNRNPM protein was also found to be differentially spliced in human AD cases compared to controls as reported in Johnson et al. [1]. In 12-month-old Trem2*R47H male mice, we did not observe a significant increase (*p* > 0.05) in overall expression of the *Hnrnpm* gene, but ISAR identified significantly increased usage of the non-coding isoform of *Hnrnpm* gene (isoform that do not contain RNA recognition motif (RRM)) and decreased usage of primary isoforms with RRM in *Hnrnpm* gene (Supplementary Fig. 4). RRM is the most abundant RNA binding domain [39] and is required for mRNA processing and splicing, suggesting a possible loss of nuclear spliceosome activity of HNRNPM protein due to differential transcript usage.

### Differentially spliced genes are predicted to have multiple binding sites of disrupted RNA binding proteins

Disruption of RNA binding proteins can potentially affect the splicing of their targets. To test this, we first investigated the binding sites of RNA binding protein RBM25 (encoded by significantly differentially expressed *Rbm25* gene in 12-month-old Trem2*R47H male mice that are associated with the spliceosome pathway) on differentially spliced genes identified by our approaches and reported in at-least one human AD splicing study. RBM25 is U1 small nuclear ribonucleoprotein with BAD domains and has been suggested to have increased aggregation in Alzheimer’s disease [40]. We identified binding sites of RBM25 protein on differentially used exons of multiple DEU genes such as *PPP1R9B, CELF4, FARS2, MACF1*, *ENO2, STXBP1* (Supplementary Table 7), and on multiple exonic regions of all DTU genes (Supplementary Table 8). Identification of binding sites of RBM25 in these differentially spliced genes suggests that significant increased expression of RBM25 protein in 12-month-old Trem2*R47H male mice may lead to the differential splicing of genes identified in our study. We could not investigate binding sites of other differentially expressed genes (*Acin1, Rp9, Snrnp27* and *Prpf38b*) enriched for spliceosomes pathways in 12-month-old Trem2*R47H male mice, as they were not present in the experimentally verified RBPmap database.

Further, ISAR identified the differential splicing of another type of RNA binding protein HNRNPM. RNA abundances of hnRNPs are altered in many types of cancer and neurodegenerative diseases, such as spinal muscular atrophy (SMA), amyotrophic lateral sclerosis (ALS), Alzheimer’s disease (AD), and frontotemporal lobe dementia (FTLD) [39]. hnRNP M proteins are an abundant group of hnRNPs that have been shown to bind avidly to poly(G) and poly(U) RNA homopolymers [41]. hnRNP M family members can induce exon skipping and promote exon inclusion, suggesting that the proteins may broadly contribute to the fidelity of splice site recognition and alternative splicing regulation [42]. Neurons are non-dividing cells and therefore need a tight regulation of mRNA homeostasis, so they are highly vulnerable to dysfunction of RNA-binding proteins (RBPs), including the hnRNPs [39]. Therefore, we hypothesized that RNA splicing factors, such as hnRNPM might be disrupted in Trem2*R47H mice due to increased usage of non-coding isoforms, which further results in splicing defects in other neuronal cell genes regulated by hnRNP M proteins.

To test this hypothesis, we investigated binding sites of differentially spliced RNA-binding proteins HNRNPM and CELF5 on differentially spliced genes (genes with differentially used exons and/or differentially transcript usages) in Trem2*R47H, as these two regulatory proteins were present in the RBPmap webserver [43]. For this study, we primarily focused on genes that are either identified as differentially spliced using both DEXSeq and ISAR tools and reported in at-least one human AD splicing studies (*Ppp1r9b, Celf4, and Fars2*) or reported in multiple human AD splicing studies and exhibit either differentially used exons or differential transcript usages (*MACF1, PPP3CA, ENO2, KIF21A, EFTUD2, STXBP1, PFN2*, and *PSMD2*) (Table 7). We assess the binding sites of the HNRNPM protein on all exons (input region included 10 bp before exon starts and 10 bp exon end position) of these selected genes.

**Table 7:** Mouse genes identified either in both approaches/multiple human AD splicing studies: Genes that are either identified as differentially spliced using both DEXSeq and ISAR tools and further reported to be differentially spliced in at-least one human AD splicing study or reported to be differentially spliced in multiple human AD splicing studies and also identified by any one of these tools in 12 months old Trem2*R47H male mice

		**Splicing Analysis Tools**		**Human AD splicing Studies**		
**SYMBOL**	**ENSEMBL**	**DEXSeq**	**ISAR**	**ROSMAP**	**TMT-Proteomics**	**Emory/Kentucky**
*Ppp1r9b*	ENSMUSG00000038976	Y	Y	-	Y	-
*Celf4*	ENSMUSG00000024268	Y	Y	-	-	Y
*Fars2*	ENSMUSG00000021420	Y	Y	-	-	Y
*Macf1*	ENSMUSG00000028649	Y	N	Y	Y	-
*Ppp3ca*	ENSMUSG00000028161	Y	N	Y	Y	Y
*Eno2*	ENSMUSG00000004267	Y	N	Y	Y	Y
*Stxbp1*	ENSMUSG00000026797	Y	N	-	Y	Y
*Kif21a*	ENSMUSG00000022629	Y	N	Y	Y	-
*Eiftud2*	ENSMUSG00000020929	Y	N	-	Y	Y
*Pfn2*	ENSMUSG00000027805	N	Y	-	Y	Y
*Psmd2*	ENSMUSG00000006998	N	Y	-	Y	Y

First, we examined the binding sites of HNRNPM on differentially used exons of DEU genes from the list (*PPP1R9B, CELF4, FARS2*, *MACF1, PPP3CA, ENO2, KIF21A, EFTUD2, and STXBP1)* and found multiple binding sites on differentially used exon in *Ppp1r9b and Fars2 gene* located at 3’end of gene (Supplementary Table 7). No binding sites of the HNRNPM protein were found in differentially used exon of rest of the selected genes. Next, we assess the binding sites on all the exons of DTU genes (*PPP1R9B, CELF4, FARS2, PFN2*, and *PSMD2*) from the list, and identified binding sites of HNRNPM protein on multiple exons of these DTU genes (Supplementary Table 8). We extended our analysis on all other DTU and DEU genes that were also reported differentially spliced in human AD studies and found binding sites of HNRNPM protein on all DTU genes except the *Clu* gene, and 10 other DEU genes (Supplementary Tables 7 and 8). Similarly, we investigated binding site of CELF5 and identified binding sites on multiple differentially spliced genes (Supplementary Tables 7 and 8). All together, these results suggest that disruption of RNA binding proteins may lead to differential splicing of other target genes.

## Discussion and conclusions

In this study, we investigated alternative gene splicing events in aging brain transcriptomes of LOAD mouse models in both sexes using state-of-the-art analytic methods. Notably, we observed more alternative gene splicing events in middle-aged *Trem2.R47H* mice that were further enriched for AD associated neuronal functions. Similar splicing alterations were observed in *Trem2* knockout mice, implying that these events are not uniquely caused by the R47H mutation and at least partially driven by the reduced *Trem2* transcript expression in our Trem2*R47H mouse model [44]. However, splicing differences appeared at a younger age in the full knockout suggesting a more severe effect in mice lacking *Trem2*. Interestingly, we did not observe a significant overlap between differential expressed genes and differential spliced genes. We further determined that differentially expressed and differentially spliced genes represent distinct biological processes associated with AD, suggesting that splicing analysis identified novel associations and uncovered functional diversity that had been missed by conventional approaches evaluating gene expression only.

Further assessment identified enrichment for neuronal cell signatures in the differentially spliced genes, while DEGs were enriched for microglial cell signatures. Since *Trem2* is expressed in microglia and not neurons, it is not immediately clear how its perturbation leads to differential splicing in neuronal genes. In our prior characterization of these models, we did not observe any significant changes in neuron or microglial cell abundances [10]. Bidirectional communication between neurons and glial cells is required for microglia housekeeping functions and preservation of neuronal homeostasis [45, 46]. Neurons express many receptors that are activated by microglia released molecules, enabling microglial control of neurotransmission [45]. For instance, growth factors secreted by microglia (NGF, BDNF) are required by neurons for proper functioning and may not be secreted at sufficient levels. Altered expression of *Trem2* gene may perturb expression of signaling ligands secreted by microglia and thus lead to aberrant splicing in neuronal genes. Expression of cytokines and growth factors secreted by microglia involved in modulation of neuronal functions did not exhibit significant transcriptomic changes in the middle-aged *Trem2.R47H* mice. However, we observed significant reduced level of BDNF in 4 months old Trem2 KO mice in cortex tissue, as well as reduced expression levels of other cytokines and growth factors (although not significantly). This further suggests that the effect of aberrant splicing is stronger and occurs at a younger age with absence of *Trem2* gene. Finally, our analysis of whole brain hemispheres precludes resolution of region-specific effects. Deeper investigation is required to properly unravel the involvement of microglia in functional synaptic regulation and understand the physiological role of microglia-neuron interactions.

There are numerous bioinformatics methods for detecting differential splicing events using different approaches. In our study, to infer differential splicing events we adopted two approaches (exon-centric and isoform-centric) [38, 47] that use same DEXSeq method [47]. Still, we did not observe significant overlap between number of genes that had been identified differentially spliced by both approaches. This reflects that different methods capture different signals and/or are biased to certain features, such as exon-centric DEXSeq is expected to be biased against small exons while isoform usage based methods can be biased against detection of differential splicing genes with many isoforms [48]. Hence, it is important to use consensus results from different approaches to infer potential differential splicing events and/or compare with human studies based on different methods [1–3]. Therefore, we have focused on differentially spliced genes that were either identified by both approaches and/or identified as differentially spliced in human AD studies [1–3]. Overall, we identified significant overlap between differentially spliced genes identified in 12-month-old *Trem2.R47H* mice and multiple human AD studies. A number of genes, including *Kif21a*,*Macf1, Eno2, Ppp3ca, Stxbp1, and Eftud2*, that were reported in more than one human AD study were also found differentially spliced in 12-month-old *Trem2.R47H* male mice. These genes represent potential candidate for further studies of disease-related changes linked to aberrant splicing.

One important finding was identification of differential isoform usages in RNA binding proteins, specifically in *Hnrnpm* gene. Family members of hnRNPs has been previously associated with Alzheimer’s disease [39] and, in particular, the gene *HNRNPM* was identified as differentially spliced in symptomatic AD cases compared to controls [1]. We observed decreased use of the primary isoform of the *hnrnpm* gene that contains an RNA recognition motif required for proper mRNA processing and splicing, suggesting that reduced usage of primary isoform of *hnrnpm* gene may cause aberrant splicing of target genes that may trigger AD associated pathogenesis resulting from TREM2 mutations or similar disease etiologies. We identified multiple binding sites of hnRNPM in alternatively spliced genes in the *Trem2.R47H* mouse, including gene markers of AD. Notably, the hnRNPM target *Ppp1r9b* was identified differentially spliced using both analytical methods (ISAR and DEXSeq), and has also been reported in human AD [1]. *Ppp1r9b* encodes a scaffold protein (known as neurabin-2 and spinophilin) that interacts with actin-filament and protein phosphatase 1a. The encoded protein acts in the nervous system to regulate spine morphology and density, synaptic plasticity, and neuronal migration [49, 50], and a reduction in its abundance was associated with increased plaque burden in a mouse model [51]. The *PPP1R9B* gene was also reported as a potential biomarker for AD in neuronal cultures exposed to Aβ [52]. Together, these findings suggest that hnRNPM-mediated changes *Ppp1r9b* encoded protein levels may disrupt synaptic transmission associated with AD pathology.

In summary, a global splicing analysis in mouse models of LOAD implicated the possible role of the *Trem2* gene in disrupting AD associated neuronal signaling process through an alternative splicing mechanism. Further studies focused on alternative splicing in specific cell types may reveal a microglial-mediated mechanism affecting alterative splicing in aging neurons. In this study, we provided a foundation for such a multi-cellular hypothesis relevant to late-onset Alzheimer’s disease, highlighting the importance of splicing analyses in approaches to evaluating gene expression levels in model systems.

## Methods

### Mouse gene expression data and data processing

All mouse data (raw fastq files and count matrices) analyzed in this study were obtained from the characterization of recently created mouse models based on late-onset AD genetics. We have provided information and Synapse ID for each fast file associated with each sample and count matrices used in this study in Supplementary Table 9. Data and experimental details are available on the AD Knowledge Portal [53] (www.synapse.org/#!Synapse:syn17095980). Briefly, RNA-Seq data were obtained from whole left hemisphere brain samples from APOE4 KI mouse, carrying a humanized version of the prominent *APOEε4* genetic risk factor for LOAD, and the Trem2*R47H mouse, carrying the R47H allele of *Trem2* gene. In addition, a mouse model expressing both human *APOEε4* and the Trem2*R47H mutation (referred to as LOAD1) [10] was used to compare the transcriptional changes in mice carrying both variants to mice carrying only a single risk allele and B6 controls (Table 8). In all cases, mice were homozygous for the engineered allele(s). Transcriptomes from left brain hemispheres were collected at 4, 8, 12, and 24 months of age from both sexes, with six replicates of each group. RNA-Seq data were processed by a parallelized and automatic bioinformatics workflow (Supplementary Fig. 1). Reads were quality trimmed and filtered using the Trimmomatic tool (v0.33) [54]. Reads passing quality filtering were mapped to the mouse genome (GRCm38.p6) augmented by human *APOE* sequence using the RNA-Seq aligner STAR (v2.5.3) [55]. Gene expression was quantified in two ways, to enable multiple analytical methods: transcript per million (TPM) using RSEM (v1.2.31) [56], and raw counts using HTSeq-count (v0.8.0) [57]. Differential expression in mouse models was assessed using the R Bioconductor package DESeq2 (v1.16.1) [47] and genes with the Benjamini–Hochberg adjusted *p*-values < 0.05 were considered as significantly differentially expressed genes.

**Table 8:** Study population. Whole brain left hemispheres were collected at 4, 8, 12, and 24 months of age from both sexes from mouse models based on late-onset AD genetics and C57BL/6J control mice

Mouse Models	4 months		8 months		12 months		24 months	
	**Male**	**Female**	**Male**	**Female**	**Male**	**Female**	**Male**	**Female**
**C57BL/6J**	12	12	6	6	6	6	7	6
**APOE4 KI**	13	12	6	6	4	5	5	6
TREM2*R47H	12	12	6	6	6	6	6	3
APOE4.TREM2*R47H	10	12	5	6	8	5	7	6

### Differential splicing analysis

Differential splicing analyses were performed using two bioinformatics tools: DEXSeq (v1.40.0), a Bioconductor package which measures differential exon usage (DEU) as a surrogate to infer differential splicing events in RNA-Seq data [31]; and IsoformSwitchAnalyzerR (ISAR) (v1.20.0), which measures differential isoform usage to identify isoform switches and predict the resulting functional consequences [38]. In DEXSeq analysis, exons with FDR < 0.1 were considered significantly differentially used. ISAR measures isoform usage via isoform fraction (IF) values, which quantify the fraction of the parent gene expression originating from a specific isoform (calculated as isoform_exp/gene_exp). Difference in isoform usage is quantified as the difference in isoform fraction (dIF). ISAR implemented DEXSeq based tests as the default way to identify isoform switches [38]. In ISAR analysis, the cutoff for change in absolute dIF was set to 0.05 to be considered potential isoform switch and switches with FDR < 0.05 were considered significant. We chose different threshold for ISAR analysis due to greater sensitivity than to DEXSeq analysis.

### Functional enrichment analysis

Functional annotations and enrichment analyses were performed using the R Bioconductor package clusterProfiler [58], with Gene Ontology terms and KEGG pathways [59–61] enrichment analyses by functions enrichGO and enrichKEGG, respectively. The function compareCluster was used to compare enriched functional categories of each gene module. The significance threshold for all enrichment analyses was set to 0.05 using Benjamini–Hochberg adjusted *p*-values.

### Cell type enrichment analysis

Different brain cell types (neurons, endothelial cell, astrocytes, microglia, oligodendrocyte precursor cells, newly formed oligodendrocytes, and myelinating oligodendrocytes) specific gene signatures were selected from an RNA-Sequencing database [32]. Fisher exact test was used to test enrichment of cell type signatures in each input gene set. Resulting p-values were corrected for multiple tests using the Benjamini–Hochberg method and the significance threshold was set to 0.1.

### Predicting binding sites of RNA-binding proteins

Binding sites of RNA-binding proteins on differentially spliced genes were predicted using RBPmap webtool [43]. We selected RNA binding proteins from RBPmap’s database of experimentally defined human/mouse motifs. Within the scope of this study, we explicitly looked for RNA-binding proteins that were either identified as differentially expressed or differentially spliced through our analyses. We used RBPmap webserver at default settings for mouse genome (GRCm38/mm10 assembly).

### *Trem2* Knockout (KO) mice transcriptomic data

RNA-Seq data (fastq files) of brain tissue samples for *Trem2* KO and respective control mice was obtained from the Gene Expression Omnibus data repository (accession number: GSE104381). Details on brain sample collection, tissue and RNA preparation, and sequencing can be found in previously published work on this data [33]. Further, we have re-processed RNA-Seq data using our RNA-Seq processing pipeline (as described above).

### Human splicing studies in AD cohort

Differentially spliced genes in the human brain associated with AD were obtained from three distinct studies. In the first study, dorsolateral prefrontal cortex (DLPFC) of 450 subjects from the Religious order Study (ROS) and the Memory and Aging Project (MAP) was deeply sequenced followed by identification of alternative splicing events related to AD by measuring differential intron usages [2]. In the second study, authors have used a new proteogenomic approach (TMT-LysC) to investigate RNA splicing events by predicting alternative exon-exon junction splicing events across symptomatic AD (*n* = 20), asymptomatic AD (*n* = 14), and control (*n* = 13) cases [1]. In the third study, authors have performed deep RNA-Sequencing of frontal cortex RNAs using sample groups from Emory university (four controls and five AD cases); and the university of Kentucky (three controls and AD cases) followed by identification of genes associated with RNA processing and splicing abnormalities in AD cases [3]. Genomic information on orthologous genes was obtained via the latest ENSEMBL build for human genome version GRCh38. All orthologous relationships between human and mouse were downloaded via BioMart [62]. Next, we performed hypergeometric test to compute significant overlaps (*p* < 0.05) between differentially spliced genes identified in each human study and each mouse model.

## Supplementary Information

**Additional file 1: Supplementary Fig. 1.** Bioinformatics workflow with each step of the splicing analysis.

**Additional file 2: Supplementary Text 1.** Documentation of each tool and command used for each step mentioned in the bioinformatics workflow.

**Additional file 3: Supplementary Fig. 2.** Differential splicing analysis in Trem2.KO mouse models. (A) Number of differentially expressed genes (DEGs) and genes with differential exon usage (DEU) in Trem2.KO mice at 4 and 8 months of age in cortex and hippocampus brain tissues. (B) Enrichment of KEGG pathways (*p* < 0.05) in DEU genes in Trem2 KO mice. (C) Cell type enrichment analysis in the gene set with differential exon usage (DEU) and differentially expressed genes (DEG) in each mouse model using Fisher exact test. Significant cell type enrichment are shown by solid dots (*p* < 0.1).

**Additional file 4: Supplementary Fig. 3.** Cell type enrichment in differentially spliced genes in human AD cohorts. Cell type enrichment analysis in the differentially spliced genes in human AD populations were computed using Fisher exact test. Solid circles represent significant enrichment (*p* < 0.1) of cell type.

**Additional file 5: Supplementary Fig. 4.** ISAR identified differential isoform usage in Hnrnpm, which encode for RNA binding protein (RBP) HNRNPM. Significant increased usage ( FDR < 0.05 was observed for non-coding isoform in 12 months old Trem2*R47H male mice compared to B6 mice.

**Additional file 6: Supplementary Table 1.** Differentially exon usage (DEU) genes identified in mouse models. The attached table depicts the differentially expressed usage genes in LOAD mouse strains compared to C7BL/6 J mice using DEXSeq tool.

**Additional file 7: Supplementary Table 2.** Enriched KEGG pathways in DEU genes in each mouse model. The attached table depicts the KEGG pathway annotations for the differentially exon usage genes in LOAD mouse strains and Trem2 KO mice compared to C7BL/6 J mice.

**Additional file 8: Supplementary Table 3.** Overlap between differentially expressed genes and differentially spliced in each mouse model. The attached tables depicts overlap between the differentially exon usage genes (DEUs) and differentially expressed genes (DEGs), and differentially isoform usage genes (DTUs) and differentially expressed genes (DEGs) in LOAD mouse strains compared to C7BL/6 J mice.

**Additional file 9: Supplementary Table 4.** Differentially exon usage genes in Trem2 KO mice. The attached table depicts the differentially expressed usage genes in Trem2 KO mice compared to C7BL/6 J mice using DEXSeq tool.

**Additional file 10: Supplementary Table 5.** Differentially isoform usage (DTU) genes identified in each mouse model. The attached table depicts the differentially expressed usage genes in LOAD mouse strains compared to C7BL/6 J mice using IsoformSwitchAnalyzeR (ISAR) tool.

**Additional file 11: Supplementary Table 6.** Enrichment of Gene ontology biological processes analyses in differentially isoform usage (DTU) genes. The attached table depicts the gene 2 ontology enrichment for the differentially isoform usage genes in Trem2*R47H male and female mice compared to age and sex-matched C7BL/6 J mice.

**Additional file 12: Supplementary Table 7.** List of differentially DEU genes with binding sites of RNA binding proteins. The attached file contains the differentially exon usage genes in Trem2*R47H male mice with binding sites for RNA-binding proteins.

**Additional file 13: Supplementary Table 8.** List of DTU genes with binding sites of RNA binding proteins. The attached file contains the differentially isoform usage genes in Trem2*R47H male mice with binding sites for RNA-binding proteins.

**Additional file 14: Supplementary Table 9.** Information about each sample and unique Synapse ID for each sample for each associated raw FASTQ files and raw gene count matrices used in this study.

## Data Availability

The B6.*APOE4.Trem2*R47H* (LOAD1) data sets are available via the AD Knowledge Portal (https://adknowledgeportal.org). The AD Knowledge Portal is a platform for accessing data, analyses, and tools generated by the Accelerating Medicines Partnership (AMP-AD) Target Discovery Program and other National Institute on Aging (NIA)-supported programs to enable open-science practices and accelerate translational learning. The data, analyses and tools are shared early in the research cycle without a publication embargo on secondary use. Data is available for general research use according to the following requirements for data access and data attribution (https://adknowledgeportal.org/DataAccess/Instructions). The results published here are in whole or in part based on data obtained from the AD Knowledge Portal. Study data were provided by the Rush Alzheimer’s Disease Center, Rush University Medical Center, Chicago. Study data were provided through the Accelerating Medicine Partnership for AD (U01AG046161 and U01AG061357) based on samples provided by the Rush Alzheimer’s Disease Center, Rush University Medical Center, Chicago. Data collection was supported through funding by NIA grants P30AG10161, R01AG15819, R01AG17917, R01AG30146, R01AG36836, U01AG32984, U01AG46152, the Illinois Department of Public Health, and the Translational Genomics Research Institute. For access to content described in this manuscript see: https://doi.org/10.7303/syn23631984. Detailed documentation reporting each tool (software name and version) and commands used to analyze the mouse data are provided in Supplementary Text 1.

## Abbreviations

AD: Alzheimer’s disease
AsymAD: Asymptomatic AD
AMP-AD: Accelerating Medicines Partnership—Alzheimer's Disease
B6: C57BL/6J
DEG: Differentially expressed genes
DEU: Differentially exon usage
DTU: Differential transcript usage
LOAD: Late-onset Alzheimer’s disease
RBP: RNA binding protein
ROS/MAP: Religious Orders Study and Memory and Aging Project
RRM: RNA recognition motif
