# Supplementary figures and images for: Differential splicing of neuronal genes in a Trem2*R47H mouse model mimics alterations associated with Alzheimer’s disease
Source: BMC Genomics. 2023 Apr 4;24:172. doi: 10.1186/s12864-023-09280-x (PMC10074678; doi:10.1186/s12864-023-09280-x)

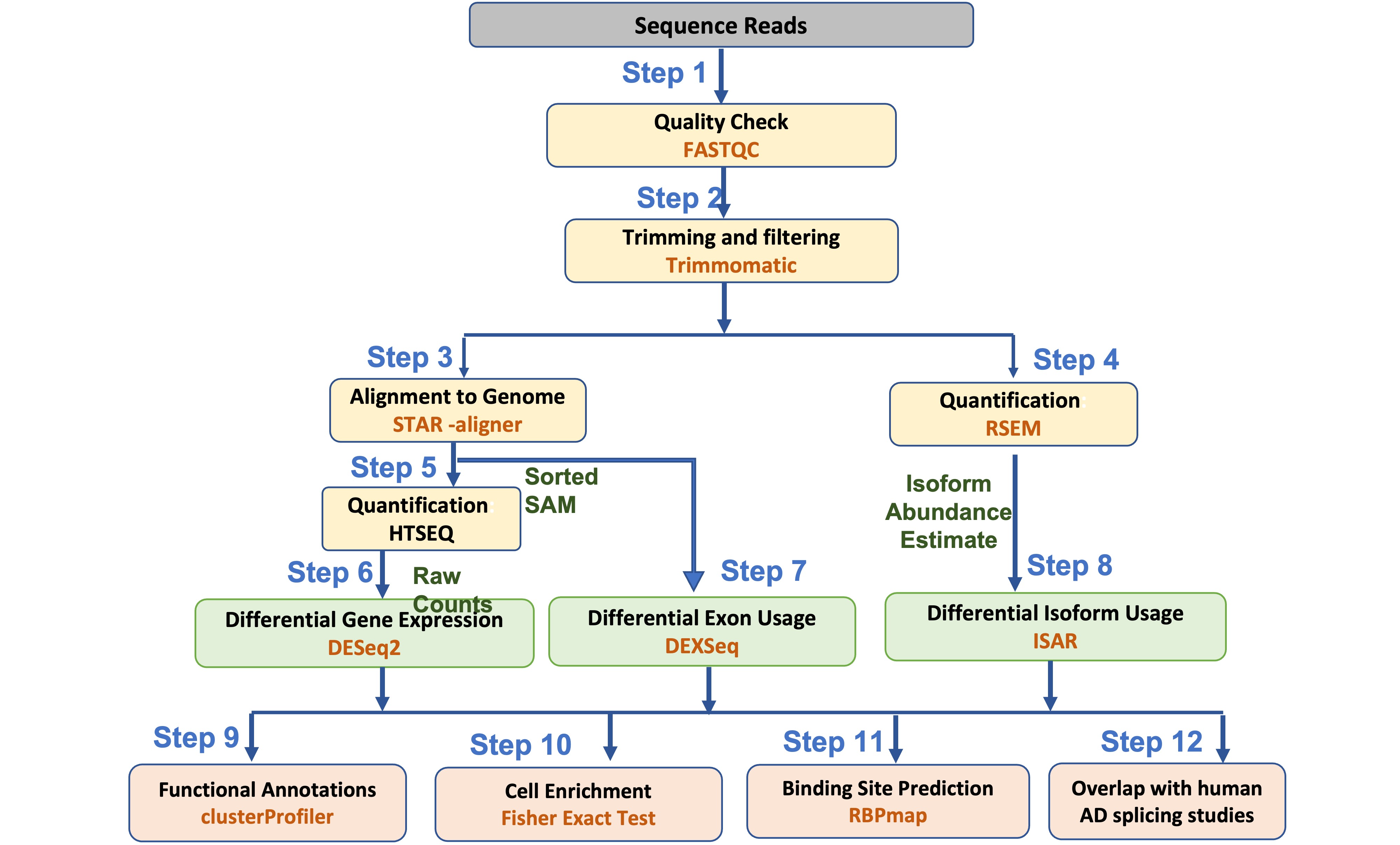

Supplement: Supplementary file 1 — Additional file 1: Supplementary Fig. 1. Bioinformatics workflow with each step of the splicing analysis. [file 12864_2023_9280_MOESM1_ESM.tiff]

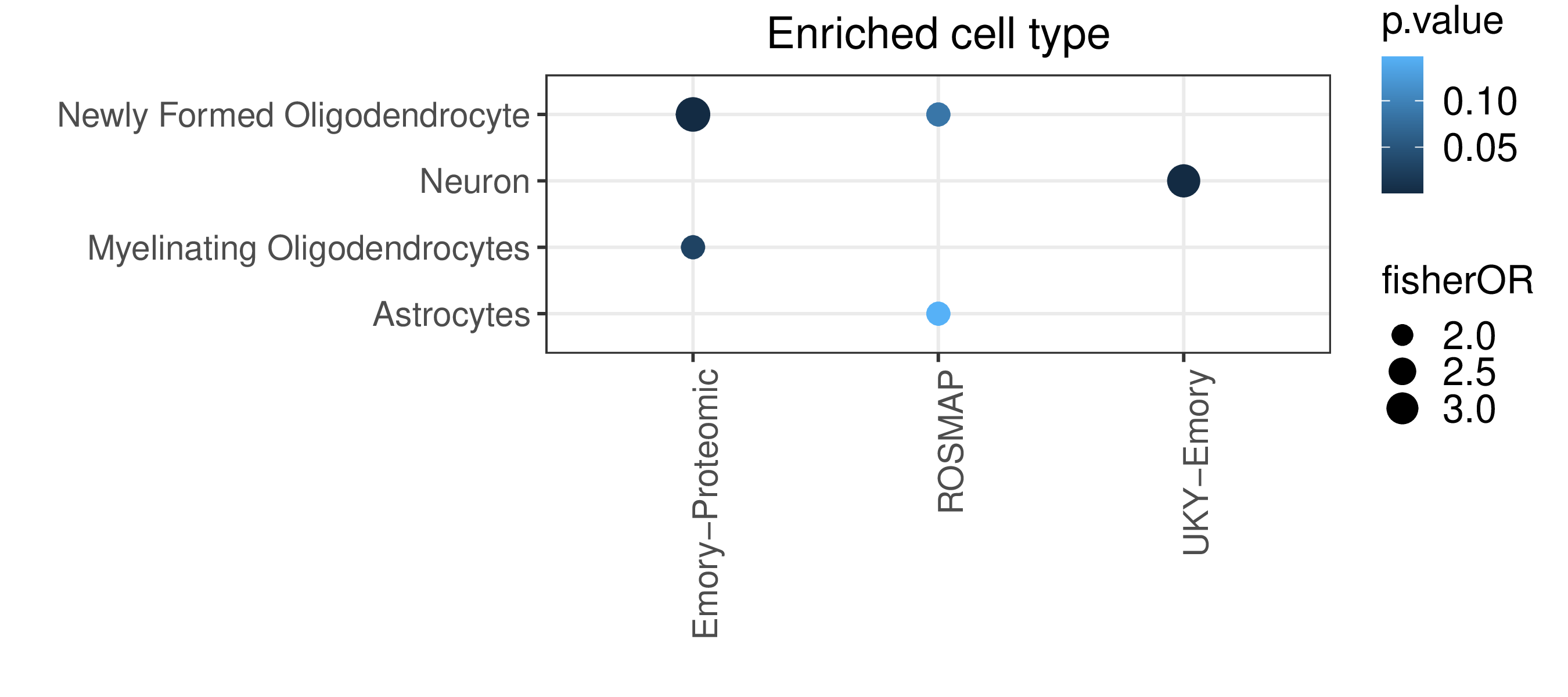

Supplement: Supplementary file 4 — Additional file 4: Supplementary Fig. 3. Cell type enrichment in differentially spliced genes in human AD cohorts. Cell type enrichment analysis in the differentially spliced genes in human AD populations were computed using Fisher exact test. Solid circles represent significant enrichment (p < 0.1) of cell type. [file 12864_2023_9280_MOESM4_ESM.tiff]

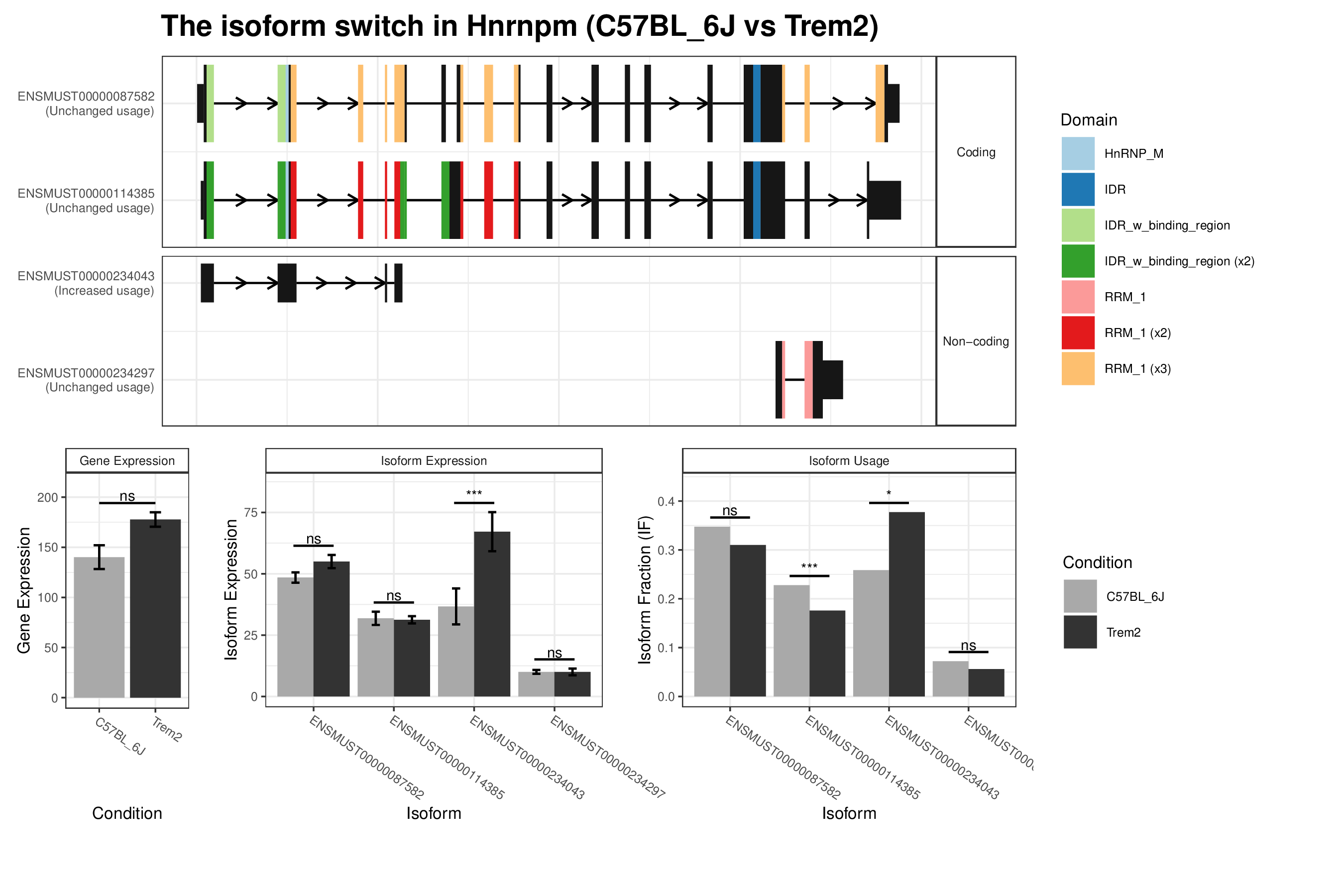

Supplement: Supplementary file 5 — Additional file 5: Supplementary Fig. 4. ISAR identified differential isoform usage in Hnrnpm, which encode for RNA binding protein (RBP) HNRNPM. Significant increased usage ( FDR < 0.05 was observed for non-coding isoform in 12 months old Trem2*R47H male mice compared to B6 mice. [file 12864_2023_9280_MOESM5_ESM.tiff]
