# Supplementary material for: Differential splicing of neuronal genes in a Trem2*R47H mouse model mimics alterations associated with Alzheimer’s disease
Source: BMC Genomics. 2023 Apr 4;24:172. doi: 10.1186/s12864-023-09280-x (PMC10074678; doi:10.1186/s12864-023-09280-x)
Supplement: Supplementary file 2 — Additional file 2: Supplementary Text 1. Documentation of each tool and command used for each step mentioned in the bioinformatics workflow. [file 12864_2023_9280_MOESM2_ESM.pdf]

Documentation of Bioinformatics Analysis Workflow

PART1: RNA-Seq Processing workflow

I have outlined version and commands of all used tools for processing paired-end fastq files from each sample in unix environment.

Step1: Quality Check using FASTQC

Version used : FastQC v0.11.3

```
fastqc R1.fq R2.fq -o outputdir
```

Step2: Trimming low quality bases using TRIMMOMATIC [1]

Java-version used : 1.7.0_79
Trimmomatic-version used : 0.33

```
java -jar trimmomatic-0.33.jar PE R1.fq R2.fq R1_paired.fq R1_unpaired.fq R2_paired.fq R2_unpaired.fq LEADING:3 TRAILING:3 SLIDINGWINDOW:4:15 MINLEN:36
```

Step3: Alignment to reference genome using STAR-Aligner [2]

STAR version used : v2.5.3
Picard version: v1.95

3.1 Create STAR Index for alignment

```
star-2.5.3  --runThreadN 4 --runMode genomeGenerate --genomeDir /path/to/genome/index/ --genomeFastaFiles /path/to/ genome/referenceGenomeFile --sjdbGTFfile /path/to/ gtfFile/referencegtfFile --sjdbOverhang 100
```

3.2 Alignment to reference genome

```
star-2.5.3 --genomeDir /path/to/genome/index/ --readFilesIn R1_paired.fq
R2_paired.fq --runThreadN 12 --outFileNamePrefix filePrefix --quantMode
GeneCounts --sjdbGTFfile /path/to/gtfFILE
```

3.3 Sorting SAM files by co-ordinates

```
java -Xms1g -Xmx4g -jar picard-v1.95/SortSam.jar INPUT= alignment_sample1.sam
OUTPUT= sorted_ sample1.sam SO=coordinate
```

3.4 Converting SAM files to BAM files

```
samtools_1.10  view -bS sorted_ sample1.sam -o sample1.out.bam
samtools_1.10  index sample1.out.bam
```

Step4: Quantification using HTSEQ [3]

Version used : HTSeq-v0.8.0

```
htseq-count -s no  -f sam sorted_sample1.sam /path/to/gtfFILE >
rawcount_sample1.txt
```

Step5: Implementation of RSEM [4] to obtain isoform abundances in each sample

Version used : rsem_v1.2.31

5.1 Create RSEM STAR Index for alignment

```
rsem_v1.2.31  rsem-prepare-reference --gtf /path/to/ genome/referencegtfFile --
star --star-path /path/to/STAR/2.5.3/bin -p 8 /path/to/
genome/referenceGenomeFile  /path/to/RSEM_star_index/
```

5.2 Alignment and Quantification using RSEM

```
rsem_v1.2.31 rsem-calculate-expression --paired-end --star --star-path
/path/to/STAR/2.5.3/bin -p 8 R1_paired.fq.gz R2_paired.fq.gz
/path/to/RSEM_star_index/
```

<u>NOTE:</u> Using above workflow and commands we obtained raw gene count, Isoform abundances and sam file for each sample. These outputs were used as input to DESeq2, ISAR and DEXSEQ tool for downstream analysis, respectively.

PART 2: Downstream Analysis

Step6: Differential gene expression analysis using DESeq2 [5]

We identified differentially expression genes (DEGs) using the R Bioconductor package DESeq2(v1.16.1).

6.1 Loading the libraries

```
library("DESeq2")
library("org.Mm.eg.db")
library("AnnottaionDbi")
library("xlsx")
```

6.2 Reading the metadata file

```
metadata <- read.csv("/path/to/ metadataFile", row.names = 1)

metadata <- meta[sort(rownames(metadata)),]
```

6.3 Reading the count data file obtained from HTSeq-count

```
rawdata <-  read.csv("/path/to/htseq_countdata_File", check.names = FALSE,
row.names = 1)

rawdata <- rawdata[,sort(colnames(rawdata))]
```

6.4 Differential Expression Analysis

After reading the raw count data and metadata, we performed the DEG analysis for each LOAD model [6] compared to sex and age-matched B6 controls, as shown below through an example.

Example:

To identify DEGs in 12 months old male TREM2 mice compared to age and sex-matched control mice, first we subset information about 12 months old male B6 and TREM2 samples from metadata file, as shown below:

```
selected.samples <- metadata[metadata$Sex=="Male" & metadata$Age==12 &
(metadata$Genotype=="Trem2" | metadata$Genotype=="C57BL/6J"),]
```

next, we implemented our **DEG()** function on selected samples, as shown below:

```
DEG(rawdata=rawdata,meta= selected.samples,include.batch=FALSE)
dseq_Trem2.M12 <- dseq_res              ##only DEGs
All_Trem2.M12 <- All_res                 ##All genes
```

We repeated step 6.4 for each mouse model for all ages and sexes and saved the results for downstream analysis.

Function used to identify DEGs in each LOAD mouse model

```
DEG  <- function(rawdata,meta, include.batch = FALSE, ref = "C57BL/6J")
{
  dseq_res <- data.frame()
  All_res <- data.frame()

  if (include.batch)
{
    cat("Including batch as covariate\n")
    design_formula <- ~ Batch + Genotype
  }
  else
{
    design_formula <- ~ Genotype
  }

  dat2 <- as.matrix(rawdata[, colnames(rawdata) %in% rownames(meta)])
```

```
  ddsHTSeq <-
  DESeqDataSetFromMatrix(countData = dat2,
                   colData = meta,
                   design = design_formula)
 ddsHTSeq <- ddsHTSeq[rowSums(counts(ddsHTSeq)) >= 10, ]
 ddsHTSeq$Genotype <- relevel(ddsHTSeq$Genotype, ref = ref)
 dds <- DESeq(ddsHTSeq, parallel = TRUE)

 res <- results(dds, alpha = 0.05)
 summary(res)
 res_sub <- subset(res[order(res$padj), ], padj < 0.05)

# Adding gene symbol to results mapping to ensemble ID using a map_function
 res_sub$symbol <- map_function.df(res_sub,"ENSEMBL","SYMBOL")
 res$symbol <- map_function.df(res,"ENSEMBL","SYMBOL")

# Adding Entrez ID to results mapping to ensemble ID using a map_function
 res_sub$EntrezGene <- map_function.df(res_sub,"ENSEMBL","ENTREZID")
 res$EntrezGene <- map_function.df(res,"ENSEMBL","ENTREZID")

 dseq_res <<- as.data.frame(res_sub[, c(7:8, 1:6)])

 All_res <<- as.data.frame(res[, c(7:8, 1:6)])

}
```

Function to map ENSEMBL ID to gene symbol and EntrezID

```
map_function.df  <-  function(x, inputtype, outputtype)
                   {
                        mapIds(org.Mm.eg.db,
                        keys = row.names(x),
                        column = outputtype,
                        keytype = inputtype,
                        multiVals = "first"
                        )
                   }
```

Step7: Differential Exon usages (DEU) analysis using DEXSeq [7]

For differential exon usage analysis, we followed the steps described in the documentation of DEXSeq (v1.40.0). (https://bioconductor.org/packages/release/bioc/vignettes/DEXSeq/inst/doc/DEXSeq.html). We ran all required steps at default parameter setting as mentioned in above documentation and briefly illustrated below:

7.1 Preparing the annotation for DEXSeq

We used python script `dexseq_prepare_annotation.py` provided with DEXSeq tool to convert reference mouse GTF file with gene models into a GFF file with collapsed gene models as required by DEXSeq, as shown below:

```
python dexseq_prepare_annotation.py referencegtfFile flatt.mm.gff
```

7.2 Counting the reads

We first converted BAM files to SAM files:

```
samtools_1.10 view /path/to/sample1.out.bam > sample1.sam
```

We used python script `dexseq_count.py` provided with DEXSeq tool to count number of reads that overlap with each of the exon counting bin for each sample, as shown below:

```
python dexseq_count.py -p yes -r pos flatt.mm.gff sorted_ sample1.sam
Sample1.txt
```

7.3 Reading the data in to R

Next, we load all required libraries and read count matrix and gff file generated from above commands as described in DEXSeq documentation.

```
library(“DEXSeq”)
library( "BiocParallel")
library(“dplyr”)
library("xlsx”)
```

```
library("tidyverse")

inDir = "/path/to/DEXCount/"
countFiles = list.files(inDir, pattern=".txt$", full.names=TRUE)
basename(countFiles)

flattenedFile = list.files(indir, pattern="gff$", full.names=TRUE)
basename(flattenedFile)
```

7.4 Prepare the sampleTable

Next step is to create a table for samples to be used to identify differential exon usage i.e., control and LOAD samples. For example, I have shown sampleTable we created for comparison between control B6 and Trem2 mice, as follows:

```
sampleTable = data.frame(
row.names = c("control1", "control2", "control3", "control4", "control5",
"control6", "TREM2.1", "TREM2.2", "TREM2.3", "TREM2.4" ,
"TREM2.5","TREM2.6"),
condition = c("control", "control", "control", "control","control","control","Trem2",
"Trem2", "Trem2",  "Trem2", "Trem2", "Trem2"))
```

7.5 Constructing an DEXSeqDataSet object

This object holds all the input data and will be used for differential exon usage analysis:

```
dxd = DEXSeqDataSetFromHTSeq(
  countFiles,
  sampleData=sampleTable,
  design= ~ sample + exon + condition:exon,
  flattenedfile=flattenedFile )
```

7.6 Testing for differential Exon usage and saving the significant results

We used the wrapper function DEXseq() for differential exon usage analysis in one command, as shown below:

```
dxr = DEXSeq(dxd,BPPARAM=MulticoreParam(workers=4))
```

7.7 subsetting significant exonic regions with a false discovery rate of 10%

```
DXR_TR_4M <- dxr[(dxr$padj) < 0.1,] %>%
                mutate(Symbol = map_sym(groupID))  %>%
                mutate(EntrezID = map_eid(groupID)) %>%
                filter(!(is.na(Symbol))) %>%
                select(GeneID=groupID, Symbol, EntrezID, ExonID=featureID,
exonBaseMean, dispersion, stat, padj)
```

where map_sym and map_eid are functions to map ENSEMBL ID to Gene symbol and EntrezID, as shown below:

```
map_sym <- function(x)
                {
        mapIds(org.Mm.eg.db,
        keys=x,
        column="SYMBOL",
        keytype="ENSEMBL",
        multiVals="first")
        }

map_eid <- function(x) {
        mapIds(org.Mm.eg.db,
        keys=x,
        column="ENTREZID",
        keytype="ENSEMBL",
        multiVals="first")
        }
```

We repeated steps 7.4 to 7.7 for each mouse model to identify differentially exon usage in all mouse models compared to age and sex-matched B6 control mice.

7.8 Saving the results obtained from DEU analysis into supplementary File 1

Finally, we saved DEXSeq results for each mouse model as shown below:

```
write.xlsx(DXR_TR_4M, file="/path/to/ Supplementary Table1.xlsx",sheetName = "Trem2_4mo_Male",append=TRUE)
```

Note: Similarly, we performed the DEXSeq analysis on sequencing data file obtained from cortex and hippocampus brain regions of 4 and 8 months old Trem2 KO mouse models compared to age and brain-region matched B6 controls [8] and appended the results in **supplementary Table 4**:

```
write.xlsx(Cortex_4mo_Male.Trem2, file="/path/to/ Supplementary Table4.xlsx",sheetName = " Cortex_4mo_Male",append=TRUE)
```

7.9 Overlap between genes with differential expression and exon usage

Next, we compared genes with differential exon usages and differential expression obtained from DESeq2 analysis and results from this analysis were appended as sheetName “Overlap_DEGs_DEU” in **supplementary Table 3.**

Step8: Identification of Isoform Switches using IsoformSwitchAnalyzeR (ISAR) [9]

We followed the steps described in the documentation of ISAR (V1.20.0) (https://bioconductor.org/packages/release/bioc/vignettes/IsoformSwitchAnalyzeR/inst/doc/IsoformSwitchAnalyzeR.html). For more detailed explanation about each step please follow above documentation.

ISAR takes isoform-level quantification, so we input isoform abundance count matrix generated from RSEM tool as input to ISAR. We ran all steps at default parameter setting as mentioned in above documentation.

8.1 Import data into R :

Loading libraries and specifying path to directory containing isoform count matrix for all mouse models.

```
library(IsoformSwitchAnalyzeR)
library(tidyverse)
library(BSgenome.Mmusculus.UCSC.mm10)

filedir <- "/path/to/isoform/countdata/"

metadata <-
read.csv("/path/to/metadata.csv",row.names=1,stringAsFactors=FALSE)
```

8.2 Selection of samples for Isoform usage analysis

Next we selected samples for analysis. For example, we selected 12 months old male Trem2 and C57BL/6J control mice to run the analysis. Users can do similarly for other comparisons.

```
sub.meta <- metadata[metadata$Sex=="M" & metadata$Age==12 &
(metadata$Genotype=="Trem2" | metadata$Genotype=="C57BL/6J"),]

myQuant <- importIsoformExpression(
  sampleVector = paste0(filedir,rownames(sub.meta),".isoforms.results"),
)
```

The result of using importIsoformExpression is a list containing both count and abundance estimates for each isoform.

8.3 Design matrix to indicating condition of each biological replicates

```
myDesign <-data.frame(
        sampleID=rownames(sub.meta),
        condition=sub.meta$Genotype)
```

8.4 Create switchAnalyzeRlist object

```
mySwitchList <- importRdata(
  isoformCountMatrix    = myQuant$counts,
  isoformRepExpression = myQuant$abundance,
  designMatrix              = myDesign,
  ignoreAfterPeriod       = TRUE,
  isoformExonAnnoation= "/path/to/gtfFILE/mm10.GRCm38.97.gtf.gz",
  isoformNtFasta           =
c("/path/to/cdnaFile/Mus_musculus.GRCm38.cdna.all.fa.gz",
"/path/to/ncrnaFile/Mus_musculus.GRCm38.ncrna.fa.gz"), showProgress = TRUE)
```

8.5 Extract Isoform Switches and Their Sequences

We implemented high-level function isoformSwitchAnalysisPart1() to identify isoform switches and writing the nucleotide and amino acid sequences to fasta files.

```
mySwitchListPart1 <- isoformSwitchAnalysisPart1(
  switchAnalyzeRlist        = mySwitchList,
  dIFcutoff                        = 0.05,
  pathToOutput               = "/path/to/output/outdir/",
  genomeObject               = Mmusculus,
  outputSequences          = TRUE,
  prepareForWebServers = FALSE
)
```

The mySwitchListPart1 returned by isoformSwitchAnalysisPart1() has been reduced to only contain genes where an isoform switch (as defined by the alpha and dIFcutoff arguments) was identified.

It also output two fasta files: one contains nucleotide sequences of isoforms and amino acid sequences of isoforms, and it is stored in specified output directory. These files were used for external sequence analysis tools such as Pfam (for prediction of protein domains), CPAT(for prediction of coding potential), IUpred2a (for prediction of Intrinsically Disordered Regions (IDR)) and SignalP (for prediction of signal peptides) as recommended in ISAR documentation at default parameter setting. Results from these tools will be used in second part of ISAR analysis.

8.6 Plot all isoform switches and their annotations

This part includes to incorporate the results from external sequence analysis, analyzing alternative splicing, predicting functional consequences and plotting individual genes with isoform switches.

```
mySwitchListPart2 <- isoformSwitchAnalysisPart2(
  switchAnalyzeRlist              = mySwitchListPart1,
  dIFcutoff                       = 0.05,
  n                               = Inf,
  removeNoncodinORFs        = FALSE,
  consequencesToAnalyze      = c( 'intron_retention', 'coding_potential',
'ORF_seq_similarity', 'NMD_status', 'domains_identified',
'signal_peptide_identified'),
  pathToCPATresultFile         = "/path/to/cpat_result.txt"), codingCutoff = 0.721,
 pathToPFAMresultFile         = "/path/to/pfam_results.txt",
 pathToIUPred2AresultFile  ="/path/to/IUpred2a_ isoform_AA.result",
  pathToSignalPresultFile     = "/path/to/SignalIP_output_protein_type.txt",
  pathToOutput                    = "/path/to /outputdir”,
  outputPlots                     = TRUE
)
```

The isoform switch plot for each gene saved into specified directory in above command. Isoform switch result obtained from this step was saved in **Supplementary Table 5**, as follows:

```
write.xlsx(mySwitchListPart2$isoformFeatures, file=”/path/to/Supplementary
Table 5/”, sheetName=”Trem2_12mo_Male”, append=TRUE)
```

We repeated steps 8.2 to 8.6 for rest of the LOAD mouse models and Isoform switch results obtained from this analysis for each LOAD mouse model were saved in **Supplementary Table 5**

8.7 Overlap between genes with differential expression and isoform usage

Next, we compared genes with differential isoform usages and differential expression obtained from DESeq2 analysis and results from this analysis were appended as sheetName "Overlap_DEGs_DTU" in **Supplementary Table 3.**

Step 9: Functional Annotations

9.1 Functional annotations of genes with differential exon usages

We performed the KEGG pathway [10-12] enrichment analysis using the R Bioconductor package clusterProfiler [13]. We applied **compareCluster()** function to perform enrichment analysis for genes with significant exon usage in each LOAD mouse model.

```
library(clusterProfiler)
library("AnnotationDbi")
library("org.Mm.eg.db")
```

First, created a list of genes with differential exon usages for each LOAD mouse model. We used EntrezId of all genes as input.

```
geneList <- list(APOE4_M4 = DXR_APOE4_4M$EID, APOE4_M8 =
DXR_APOE4_8M$EID ,…………………………..…… ,Trem2_F24 = DXR_TR_24F$EID)
```

Next, we implemented compareCluster() function, as shown below:

```
keggterm <- compareCluster(geneCluster = geneList, fun = "enrichKEGG",organism
= 'mmu')
```

Results were plotted as shown in figure 1 using dotplot function of clusterProfiler as follows:

```
dotplot(keggterm.ALL,font.size=13,showCategory=15,title="KEGG Pathway
enrichment across mouse models")
```

Functional annotation result was saved in tabular format in supplementary File 2:

```
write.xlsx(keggterm.ALL, file="/path/to /Supplementary Table2.xlsx",sheetName = "LOAD Models")
```

similarly, we performed the KEGG pathway [10-12] enrichment analysis for genes with significant exon usage in cortex and hippocampus brain regions of 4 and 8 months old Trem2 KO mouse models and appended the results in supplementary Table 2:

```
write.xlsx(keggterm.ALL, file="/path/to /Supplementary Table2.xlsx",sheetName = "Trem2 KO",append=TRUE)
```

9.2 Functional Annotation of DTU genes in 12months old Trem2 mice

We used following function to perform gene ontology analysis for genes with differential isoform usage in 12 months old Trem2 mice:

```
ENRICHGO <-
  function(x,universe, n = 2) {
    GO.term <- enrichGO(
      gene = x,
      OrgDb = org.Mm.eg.db,
      ont = "BP",
      pvalueCutoff = 0.05,
      pAdjustMethod = "BH",
      readable = TRUE
    )
    GO.term@result <- GO.term@result[GO.term@result$Count > n, ]
    GO.term
  }
```

#12 Months old male Trem2 mice

Extracting isoform results of male Trem2 mice from saved results at step 8.6;

```
dat <- results.ISAR$Trem2.12M.Male
```

converting Ensembl id of genes with differential isoform usage to Entrez id and storing it in a vector;

```
dat.EID.male <- as.character(as.data.frame(map_eid(unique(dat
$isoformFeatures$gene_id)))[,1])
```

Implemented to ENRICHGO function to identify enriched GO processes;

```
GOterm.male.Trem2 <- ENRICHGO(dat.EID.male, 2)
```

Saving the GO annotation results in **supplementary Table 6**;

```
write.xlsx(GOterm.male.Trem2, file="/path/to/Supplementary Table 6 ",
sheetName = "Male")
```

12 Months old female Trem2 mice

Extracting isoform results of female Trem2 mice from saved results at step 8.6:

```
dat <- results.ISAR$Trem2.12M.Female
```

converting Ensembl id of genes with differential isoform usage to Entrez id and storing it in a vector;

```
dat.EID.female <-
as.character(as.data.frame(map_eid(unique(dat$isoformFeatures$gene_id)))[,1])
```

Implemented to ENRICHGO function to identify enriched GO processes;

```
GOterm.female.Trem2 <- ENRICHGO(dat..EID.female, 2)
```

Saving the GO annotation results in **supplementary Table 6**;

```
write.xlsx(GOterm.female.Trem2,file="/path/to/Supplementary Table 6 ",
sheetName = "Female",append=TRUE)
```

Step 10: Cell type enrichment

We used Different brain cell types (neurons, endothelial cell, astrocytes, microglia, oligodendrocyte precursor cells, newly formed oligodendrocytes, and myelinating oligodendrocytes) specific gene signatures were selected from an RNA-Sequencing database [14].

Fisher exact test was used to test enrichment of cell type signatures in each input gene set (differentially expressed genes, genes with differential exon usage and genes with differential isoform usage). P values and odd ratios were calculated as shown below:

```
pval <- fisher.test(matrix(c(A,B,C,D),nrow=2,ncol=2),alternative="greater")$p.value
```

```
OR <- fisher.test(matrix(c(A,B,C,D),nrow=2,ncol=2),alternative="greater")$estimate
```

Where, A implies number of microglial genes in DEU genes in given LOAD model for example in 12 months old male Trem2; B implies number of microglial genes in database minus A; C implies other DEU genes except microglial gene in given LOAD model; and D implies total genes in database minus B & C.

Step 11: Binding Site Prediction using RBPmap [15]

We used webserver of RBPmap webserver (https://rbpmap.technion.ac.il). We used RBPmap webserver at default settings for mouse genome (GRCm38/mm10 assembly). We input co-ordinates of exonic region of differentially spliced genes as listed in supplementary table 7 and 8, which were obtained from results from DEU and DTU analysis. Results from RBPmap were downloaded and appended to supplementary table 7 and 8 for binding sites of RBM25, HNRNPM and CELF5.

Step 12: Overlap with human AD splicing studies

We performed hypergeometric test to compute significant overlaps between differentially spliced genes identified in each human study and mouse models, as shown below:

```
Hmgenes  <- intersect (Hsgenes, mmgenes)

pvalue <- phyper(q,m,n,k,lower.tail = FALSE)
```

where, Hsgenes refers to total genes identified in human AD splicing studies with mouse orthologs; mmgenes refers to total differentially spliced genes in given mouse model ; Hmgenes refers to total overlapped genes between mouse model and human AD splicing study; and

q <- Hmgenes - 1
m <- Hsgenes
n <- total mouse genes with human orthologs - m
k <- mmgenes

1. Bolger, A.M., M. Lohse, and B. Usadel, *Trimmomatic: a flexible trimmer for Illumina sequence data.* Bioinformatics, 2014. **30**(15): p. 2114-2120.
2. Dobin, A., et al., *STAR: ultrafast universal RNA-seq aligner.* Bioinformatics, 2012. **29**(1): p. 15-21.
3. Anders, S., P.T. Pyl, and W. Huber, *HTSeq—a Python framework to work with high-throughput sequencing data.* Bioinformatics, 2015. **31**(2): p. 166-169.
4. Li, B. and C.N. Dewey, *RSEM: accurate transcript quantification from RNA-Seq data with or without a reference genome.* BMC Bioinformatics, 2011. **12**(1): p. 323.
5. Love, M.I., W. Huber, and S. Anders, *Moderated estimation of fold change and dispersion for RNA-seq data with DESeq2.* Genome Biology, 2014. **15**(12): p. 550.
6. Kotredes, K.P., et al., *Uncovering Disease Mechanisms in a Novel Mouse Model Expressing Humanized APOEε4 and Trem2*R47H.* Frontiers in Aging Neuroscience, 2021. **13**.
7. Anders, S., A. Reyes, and W. Huber, *Detecting differential usage of exons from RNA-seq data.* Genome research, 2012. **22**(10): p. 2008-2017.
8. Carbajosa, G., et al., *Loss of Trem2 in microglia leads to widespread disruption of cell coexpression networks in mouse brain.* Neurobiology of aging, 2018. **69**: p. 151-166.
9. Vitting-Seerup, K. and A. Sandelin, *IsoformSwitchAnalyzeR: analysis of changes in genome-wide patterns of alternative splicing and its functional consequences.* Bioinformatics, 2019. **35**(21): p. 4469-4471.
10. Kanehisa, M., *Toward understanding the origin and evolution of cellular organisms.* Protein Sci, 2019. **28**(11): p. 1947-1951.
11. Kanehisa, M., et al., *KEGG for taxonomy-based analysis of pathways and genomes.* Nucleic Acids Res, 2023. **51**(D1): p. D587-D592.
12. Kanehisa, M. and S. Goto, *KEGG: kyoto encyclopedia of genes and genomes.* Nucleic Acids Res, 2000. **28**(1): p. 27-30.

13. Yu, G., et al., *clusterProfiler: an R Package for Comparing Biological Themes Among Gene Clusters.* OMICS : a Journal of Integrative Biology, 2012. **16**(5): p. 284-287.
14. Zhang, Y., et al., *An RNA-Sequencing Transcriptome and Splicing Database of Glia, Neurons, and Vascular Cells of the Cerebral Cortex.* The Journal of Neuroscience, 2014. **34**(36): p. 11929.
15. Paz, I., et al., *RBPmap: a web server for mapping binding sites of RNA-binding proteins.* Nucleic acids research, 2014. **42**(Web Server issue): p. W361-W367.
